# Long-term autophagy is sustained by activation of CCTβ3 on lipid droplets

**DOI:** 10.1038/s41467-020-18153-w

**Published:** 2020-09-08

**Authors:** Yuta Ogasawara, Jinglei Cheng, Tsuyako Tatematsu, Misaki Uchida, Omi Murase, Shogo Yoshikawa, Yuki Ohsaki, Toyoshi Fujimoto

**Affiliations:** 1 Laboratory of Molecular Cell Biology, Research Institute for Diseases of Old Age, Juntendo University Graduate School of Medicine, 2-1-1 Hongo, Bunkyo Tokyo, 113-8421 Japan; 2 Department of Anatomy and Molecular Cell Biology, Nagoya University Graduate School of Medicine, 65 Tsurumai, Showa Nagoya, 466-8550 Japan

**Keywords:** Autophagy, Autophagosomes, Membrane lipids, Phospholipids

## Abstract

Macroautophagy initiates by formation of isolation membranes, but the source of phospholipids for the membrane biogenesis remains elusive. Here, we show that autophagic membranes incorporate newly synthesized phosphatidylcholine, and that CTP:phosphocholine cytidylyltransferase β3 (CCTβ3), an isoform of the rate-limiting enzyme in the Kennedy pathway, plays an essential role. In starved mouse embryo fibroblasts, CCTβ3 is initially recruited to autophagic membranes, but upon prolonged starvation, it concentrates on lipid droplets that are generated from autophagic degradation products. Omegasomes and isolation membranes emanate from around those lipid droplets. Autophagy in prolonged starvation is suppressed by knockdown of CCTβ3 and is enhanced by its overexpression. This CCTβ3-dependent mechanism is also present in U2OS, an osteosarcoma cell line, and autophagy and cell survival in starvation are decreased by CCTβ3 depletion. The results demonstrate that phosphatidylcholine synthesis through CCTβ3 activation on lipid droplets is crucial for sustaining autophagy and long-term cell survival.

## Abstract (web summary)

The source of phospholipids to generate autophagosomal membranes, particularly after prolonged starvation, is not well characterized. Here, the authors show that CCTβ3, the rate limiting enzyme in phosphatidylcholine synthesis, is activated on lipid droplets and sustains long-term autophagy.

## Introduction

Macroautophagy (hereafter referred to as autophagy) is a major catabolic process that degrades cellular components in the lysosome. Upon induction of autophagy, membrane sacs called isolation membranes form and elongate to enclose various contents, and become double-membrane structures called autophagosomes. Autophagosomes fuse with lysosomes for degradation of the engulfed material^1,2^.

Much progress has been made in characterizing the autophagy protein machinery^2–5^. The location of autophagosome formation has also been studied extensively^6–13^. However, much less is known about the source of phospholipids forming the autophagosomal membrane^14^. Phospholipids in existing organelle membranes may be used directly, e.g., by being transported by Atg9-positive vesicles, but they may not be sufficient to produce autophagosomes in mammalian cells, which are double membranes that are 1 μm or larger in diameter and are generated in many copies. Thus, we hypothesized that de novo phospholipid synthesis may be involved in autophagosome formation, especially when autophagy continues for a long time.

To test this idea, we investigate the contribution of de novo-synthesized phosphatidylcholine (PC), the major phospholipid in mammalian cellular membranes. In most cell types, PC is synthesized predominantly by the CDP-choline pathway (Kennedy pathway) in three steps: (1) choline phosphorylation by choline kinase; (2) conjugation of CTP to phosphocholine by CTP:phosphocholine cytidylyltransferase (CCT); and (3) PC synthesis from CDP-choline and diacylglycerol (DAG) by choline/ethanolamine phosphotransferase1 (CEPT1) and choline phosphotransferase1 (CHPT1). The CCT-catalyzed CDP-choline synthesis is the rate-limiting step that defines the PC synthetic rate^15,16^.

We find that de novo-synthesized PC is incorporated into autophagosomes in mouse embryo fibroblasts (MEFs). In accordance with this, a minor isoform of CCT, CCTβ3, is recruited to autophagic membranes after a short starvation (e.g., 1 h). As starvation is prolonged (e.g., 8 h), CCTβ3 is recruited to lipid droplets (LDs) generated from autophagic digests^17,18^, and autophagic membranes emerge from the vicinity of those LDs. The CCTβ3 expression level is correlated with the autophagic activity only in cells starved for an extended period of time, and depletion of CCTβ3 in an osteosarcoma cell line, U2OS, reduces cell survival significantly. These results indicate that activation of CCTβ3 on autophagy-derived LDs is crucial for de novo synthesis of PC, which is used for autophagic membrane formation and thereby sustains autophagy for a long time. CCTβ3 may be targeted to suppress prolonged autophagy in cancer cells in vivo.

## Results

### De novo-synthesized PC is incorporated into autophagic membranes

**Fig. 1:**
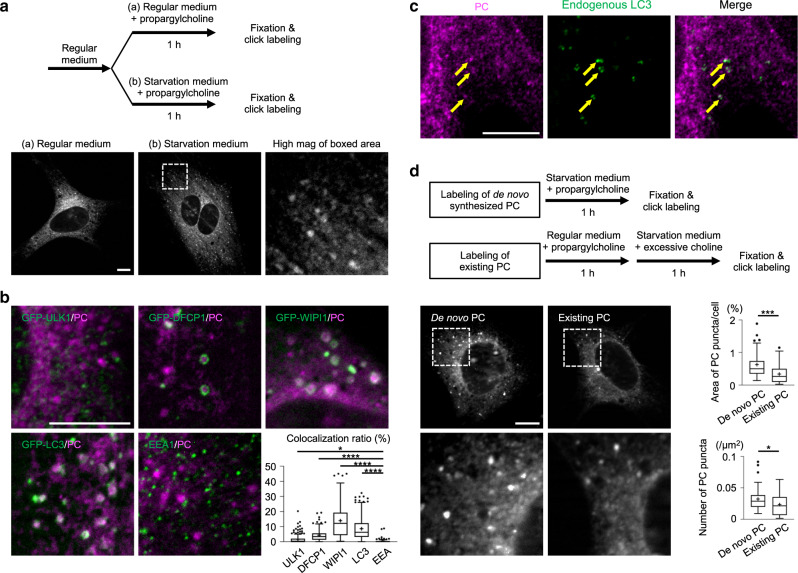
De novo-synthesized PC and autophagic membranes. **a** Labeling of de novo-synthesized PC. MEFs were cultured for 1 h with 0.25 mM propargylcholine in regular medium (a) or in starvation medium (b), fixed, and reacted with Cy3-azide for fluorescence microscopy. Bar, 10 μm. **b** De novo-synthesized PC and GFP-tagged Atg proteins in MEFs starved for 1 h. PC (magenta) and Atg proteins (green): GFP-ULK1, GFP-DFCP1, GFP-WIPI1, and GFP-LC3. Endogenous EEA1 is labeled as a control. Bar, 10 μm. The box plot shows the ratio of Atg protein (and EEA1) pixels colocalizing with PC puncta. Pooled data from three independent experiments (*n* > 130); *p = 0.0042, ****p < 0.0001 (two-tailed Mann–Whitney test). **c** Double labeling of de novo-synthesized PC (magenta) and endogenous LC3 (green) in MEFs starved for 1 h. Bar, 10 μm. **d** Labeling of existing PC. MEFs were cultured for 1 h with 0.25 mM propargylcholine in regular medium and then for the next 1 h with 5 mM choline in starvation medium. Bar, 10 μm. The total area and the number of PC puncta in a cell were measured. Pooled data from three independent experiments (*n* = 40); *p = 0.0367, ***p = 0.0002 (two-tailed Mann–Whitney test). In box plots, the center line indicates the median, box boundaries indicate the 25th and 75th percentiles, whiskers are Tukey-type, and the average is marked as +. Source data are provided as a Source data file.

De novo-synthesized choline-containing phospholipids were labeled by culturing MEFs with propargylcholine (0.25 mM) for 1 h, followed by fixation and conjugation of fluorescent azide using the click reaction^19^ (Supplementary Fig. 1A). Propargylcholine can be incorporated into sphingomyelin and lysophosphatidylcholine as well as PC, but the label obtained by the above protocol derives largely from PC^19^, and will be referred to as PC label hereafter. In MEFs cultured in regular medium, the PC label was distributed in the ER and mitochondria as described^19^ (Fig. 1a, Supplementary Fig. 1B). Notably, when MEFs were cultured for 1 h with propargylcholine in amino acid-free starvation medium, i.e., Earle’s balanced salt solution (EBSS), punctate labels were observed (Fig. 1a). The PC puncta frequently colocalized with autophagy-related (Atg) proteins: GFP-Unc-51-like kinase-1 (GFP-ULK1), GFP-double FYVE domain-containing protein 1 (GFP-DFCP1), GFP-WD repeat phosphoinositide-interacting protein 1 (GFP-WIPI1), and GFP-microtubule-associated protein light chain 3 (GFP-LC3) (Fig. 1b). The colocalization ratios of PC puncta and each Atg protein are relatively low. However, considering that respective Atg proteins represent autophagic membranes of different stages^20^ and thus do not colocalize extensively with each other (Supplementary Fig. 1C), a majority of PC puncta are thought to be distributed in autophagic membranes.

The punctate PC labeling colocalizing with GFP-LC3 was also observed in MEFs that were labeled with as little as 2.5 μM propargylcholine (Supplementary Fig. 1D). Endogenous LC3 in MEFs also colocalized with PC puncta (Fig. 1c). Formation of PC puncta and their colocalization with LC3 were similarly observed in other cell types (Supplementary Fig. 1E). The results indicated that autophagic membranes incorporate de novo-synthesized PC.

Besides de novo-synthesized PC, PC existing in organelle membranes may also be used for autophagic membranes. To examine the extent to which PC in organelle membranes are used in autophagic membrane formation, MEFs were cultured with propargylcholine for 1 h in regular medium to label PC, followed by incubation for the next 1 h in starvation medium containing excessive (5 mM) choline. PC puncta also formed by this protocol, although their total area and number were significantly smaller than those observed in cells incubated for 1 h with propargylcholine in starvation medium (Fig. 1d). The result was essentially the same when the choline concentration was equalized by adding 62 μM choline to starvation medium (Supplementary Fig. 1F), refuting the possibility that the absence of choline in starvation medium caused the difference. These results indicated that PC existing in organelle membranes may also be used for autophagic membrane formation, but at a lower amount than de novo-synthesized PC.

**Fig. 2:**
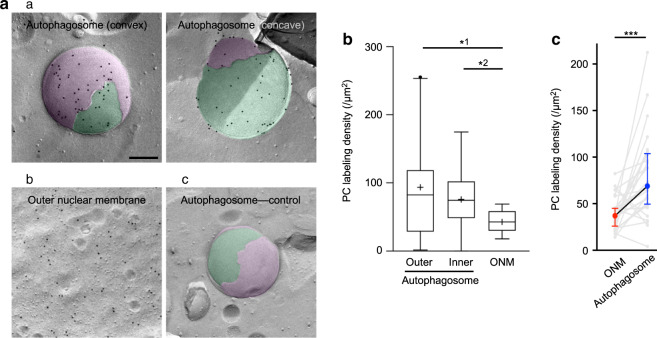
De novo-synthesized PC observed by freeze-fracture EM. **a** De novo-synthesized PC labeling. MEFs cultured for 1 h with 0.25 mM propargylcholine in starvation medium were quick-frozen and processed for EM. (a) The convex and concave fracture faces of autophagosome. Cytoplasmic leaflet (green), non-cytoplasmic leaflet (pink). See Supplementary Fig. 2B for schematic diagram of autophagosomes in freeze-fracture EM. (b) The cytoplasmic leaflet of the ONM. (c) A negative control. Copper sulfate was omitted from the click reaction solution. Bar, 0.2 μm. **b** PC labeling density. The labels of cytoplasmic and non-cytoplasmic leaflets are combined. The center line indicates the median, box boundaries indicate the 25th and 75th percentiles, whiskers are Tukey-type, and the average is marked as +. Pooled data obtained in three independent experiments. *^1^*p* = 0.0203, *^2^*p* = 0.0103 (unpaired two-tailed *t*-test). The total number of areas measured: 49 (autophagosome) and 14 (ONM). The total area measured: 13.11 μm^2^ (autophagosome) and 23.71 μm^2^ (ONM). The labeling density in respective membrane leaflets is shown in Supplementary Fig. 2C. **c** Comparison of PC labeling density in the same cell. Gray lines connect the labeling density in the autophagosome and the ONM in a cell. The medians and the 25th and 75th percentiles are shown by colored circles and error bars. Pooled data obtained in three independent experiments (*n* = 23). ***p = 0.0003 (two-tailed Wilcoxon signed-rank test). See Supplementary Fig. 2E for a representative set of micrographs that are used for this analysis. Source data are provided as a Source data file.

The above result showed that autophagic membranes incorporate de novo-synthesized PC, but it was not clear whether they do so more than the ER membrane, where PC is synthesized, because double membranes of isolation membranes/autophagosomes would emit stronger fluorescence than single membranes of the ER would, even when the signal per unit area of the membrane is similar. Thus, we used PC labeling in freeze-fracture replica electron microscopy (EM)^21^ (Supplementary Fig. 2A), which can quantify the labeling intensity in individual membranes. In this method, autophagosomes are observed as intramembrane particle-deficient double membranes^22^ (Supplementary Fig. 2B). The PC labeling occurred symmetrically in both membrane leaflets (Supplementary Fig. 2C), and it was significantly denser in autophagosomes than in the outer nuclear membrane (ONM), a proxy of the ER (Fig. 2a, b, Supplementary Fig. 2D). The difference of labeling intensity between autophagosomes and the ONM was also significant when those in the same cell were compared (Fig. 2c, Supplementary Fig. 2E). In contrast, the ER exit site, marked by GFP-Sec23, did not overlap with PC puncta, suggesting that de novo-synthesized PC is not enriched in COPII vesicles (Supplementary Fig. 2F). These results indicated that authophagic membranes incorporate newly synthesized PC at a higher density than the general ER membrane.

### CCTβ3 is recruited to autophagic membranes upon starvation

**Fig. 3:**
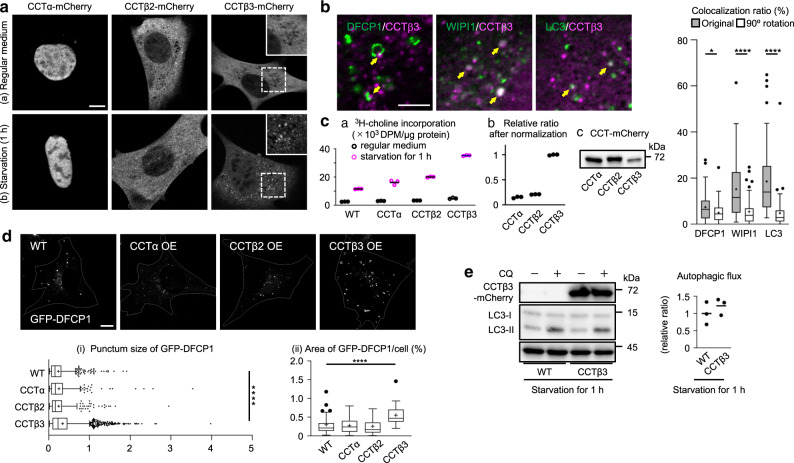
CCT isoforms in MEFs starved for 1 h. **a** Distribution of mCherry-tagged CCT isoforms in MEF. (a) Regular medium. (b) Starvation for 1 h. Bar, 10 μm. **b** Distribution of CCTβ3-mCherry (magenta) and Atg proteins (green) in MEFs starved for 1 h. Bar, 5 μm. The colocalization ratios of Atg proteins and CCTβ3-mCherry in the original picture (gray bar) and in the picture after 90° clockwise rotation of the green image (white bar) are shown. Pooled data obtained in three independent experiments (*n* = 60); *p = 0.0319, ****p < 0.0001 (two-tailed Mann–Whitney test). **c** PC synthetic activity. ^3^H-choline was added to medium 30 min before harvest. (a) MEFs with or without CCT overexpression cultured in regular medium or starvation medium for 1 h. (b) The result in starvation medium is normalized by the expression level of CCT isoforms, estimated by the CCT-mCherry band intensity in western blotting (c). A representative result of three independent experiments. **d** The effect of CCT overexpression on GFP-DFCP1 in MEFs starved for 1 h. Bar, 10 μm. (i) The punctum size. The number of puncta counted: 578 (WT), 486 (CCTα), 481 (CCTβ2), and 2320 (CCTβ3). (ii) The total area in a cell. The number of cells counted: 90. Pooled data from three independent experiments. ****p < 0.0001 (two-tailed Mann–Whitney test). **e** The effect of CCTβ3 overexpression on the autophagic flux estimated by western blotting of LC3. MEFs were cultured for 1 h in starvation medium, with or without the addition of 20 μM chloroquine (CQ) for the last 30 min. A representative result of three independent experiments. In box plots, the center line indicates the median, box boundaries indicate the 25th and 75th percentiles, whiskers are Tukey-type, and the average is marked as +. Source data are provided as a Source data file.

In MEFs that were cultured in regular medium, all three CCT isoforms showed diffuse distribution in the nucleus (CCTα) or in the cytoplasm (CCTβ2, CCTβ3), but after 1 h of starvation, only CCTβ3 showed punctate distribution (Fig. 3a) (The nomenclature of mouse CCTβ isoforms follows that of the first report^23^; please note that CCTβ2 and CCTβ3 are annotated as isoform 1 and 2, respectively, in the NCBI database [https://www.ncbi.nlm.nih.gov/protein]). This redistribution also occurred when autophagy was induced by EBSS containing 62 μM choline or by 0.25 μM Torin1 in regular medium (Supplementary Fig. 3A). The CCTβ3 puncta colocalized with the puncta of GFP-DFCP1, GFP-WIPI1, and GFP-LC3 (Fig. 3b), indicating that CCTβ3 was recruited to autophagic membranes. Endogenous LC3 also showed colocalization with CCTβ3 in starved MEFs (Supplementary Fig. 3B).

Overexpression of CCT isoforms (α, β2, or β3) in MEFs increased the PC synthetic activity only modestly in regular medium, but after starvation for 1 h, CCTβ3 overexpression enhanced the PC synthetic activity to a greater extent than that of CCTα or CCTβ2 (Fig. 3c). Overexpression of CCTβ3, but not of CCTα or CCTβ2, also increased the size and the total area of GFP-DFCP1 puncta, representing omegasomes^6^ (Fig. 3d), but the effect on the autophagic flux was minimal (Fig. 3e). These results indicate that CCTβ3 recruited to autophagic membranes activates PC synthesis, inducing omegasome enlargement, but is not sufficient to upregulate autophagy significantly.

### CCTβ3 is crucial for autophagy in prolonged starvation

**Fig. 4:**
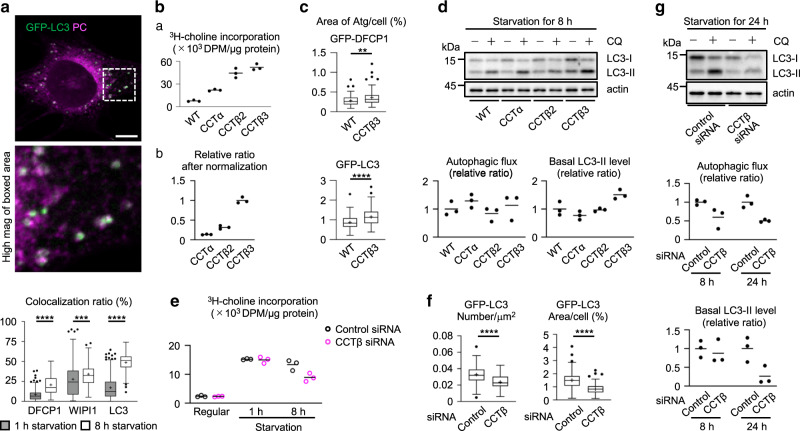
PC and CCT isoforms in MEFs starved for 8–24 h. **a** Colocalization of de novo-synthesized PC (magenta) and GFP-tagged Atg proteins (green). MEFs were cultured for 8 h in starvation medium, to which 0.25 mM propargylcholine was added for the last 1 h. Bar, 10 μm. The colocalization ratio was obtained as in Fig. 1b. Pooled data obtained in three independent experiments (*n* = 90); ***p = 0.0004, ****p < 0.0001 (two-tailed Mann–Whitney test). **b** PC synthetic activity. MEFs overexpressing CCT isoforms were cultured for 8 h in starvation medium. ^3^H-choline was added to the medium for the last 30 min. The result in (b) is normalized by the expression level of CCT isoforms, as in Fig. 3c. A representative result of three independent experiments. **c** The effect of CCTβ3 overexpression on the total area of GFP-DFCP1 and GFP-LC3 puncta in a cell. Pooled data obtained in three independent experiments (*n* = 90); **p = 0.0020, ****p < 0.0001 (two-tailed Mann–Whitney test). **d** The effect of CCT overexpression on LC3. MEFs were cultured for 8 h in starvation medium. A representative result of three independent experiments. **e**–**g** The effect of CCTβ knockdown. MEFs transfected with control siRNA and CCTβ siRNA were compared. **e** PC synthetic activity. MEFs were cultured in regular medium, in starvation medium for 1 h, or in starvation medium for 8 h. ^3^H-choline was added for the last 30 min. A representative result of two independent experiments. **f** The number and the total area of GFP-LC3 puncta. Pooled data obtained in three independent experiments (*n* = 90); ****p < 0.0001 (two-tailed Mann–Whitney test). **g** Western blotting of LC3. MEFs were cultured for 8 or 24 h in starvation medium. A representative result of three independent experiments. In box plots, the center line indicates the median, box boundaries indicate the 25th and 75th percentiles, whiskers are Tukey-type, and the average is marked as +. Source data are provided as a Source data file.

We hypothesized that existing PC is gradually exhausted when starvation is prolonged and the relative importance of de novo PC synthesis in autophagy will increase as a result. Consistent with this idea, puncta of de novo-synthesized PC showed more extensive colocalization with Atg proteins in MEFs starved for 8 h than did those starved for 1 h (Fig. 4a). PC puncta colocalization with GFP-DFCP1 appears to be less frequent than that with GFP-LC3 and GFP-WIPI1, but this apparent difference occurs because PC was mainly incorporated into the insect net-like portion of omegasomes, which emits weaker GFP-DFCP1 fluorescence than the ring portion, where LC3 localizes^6^ (Supplementary Fig. 4A). Moreover, in MEFs starved for 8 h, CCTβ3 overexpression enhanced PC synthetic activity to a larger extent than at 1 h (Fig. 4b; see Fig. 3c for comparison), and also increased the total area of GFP-DFCP1 and GFP-LC3 (Fig. 4c). The average size of GFP-DFCP1 puncta was also increased (Supplementary Fig. 4B). In accordance with the increase of the total GFP-LC3 area, the basal LC3-II level was increased by overexpression of CCTβ3, but not by overexpression of CCTα or CCTβ2 (Fig. 4d).

Consistent with the results of overexpression, CCTβ knockdown suppressed the PC synthetic activity in MEFs starved for 8 h, whereas it did not affect PC synthetic activity in MEFs cultured in regular medium or in those starved for 1 h (Fig. 4e). CCTβ knockdown also decreased the number and the total area of GFP-LC3 puncta (Fig. 4f), the autophagic flux, and the basal LC3-II level (Fig. 4g) in MEFs that were starved for 8 or 24 h. The autophagic deficiency was reproduced by transfection of another siRNA for CCTβ (Supplementary Figs. 4C, D) and rescued by re-expression of wild-type CCTβ3, but not by CCTβ2 or CCTβ3 mutants defective in the catalytic activity, CCTβ3(H59G)^24^ and CCTβ3(K92A)^25^, or membrane binding, CCTβ3(1-206)^26^ (Supplementary Fig. 4E). In contrast, the number of GFP-ULK1 puncta did not decrease by CCTβ knockdown, indicating that early stages of autophagy are not affected (Supplementary Fig. 4F). These results suggest that PC synthesis increased by CCTβ3 activation becomes more important for autophagy as starvation is prolonged and is probably involved in the formation of isolation membranes and autophagosomes.

### CCTβ3 is recruited to LDs after prolonged starvation

**Fig. 5:**
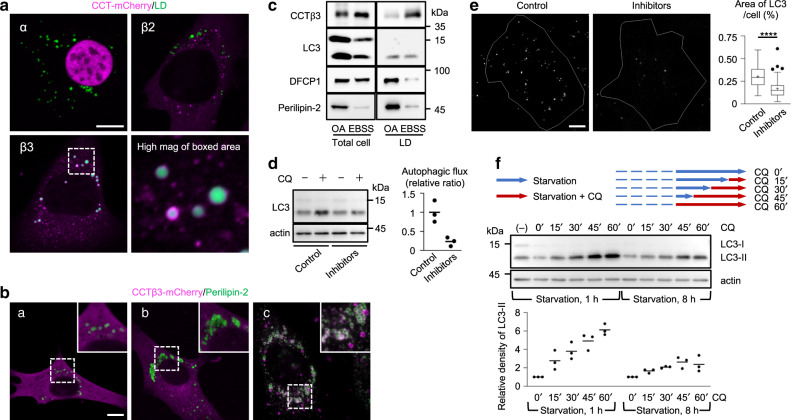
CCTβ3 in MEFs. **a** Distribution of mCherry-tagged CCT isoforms (magenta) and LDs (green) in MEFs starved for 8 h. Bar, 10 μm. **b** Distribution of CCTβ3-mCherry (magenta) and perilipin-2 (green) in MEFs. Bars, 10 μm. (a) Regular medium containing OA for 2 days. (b) Regular medium containing OA for 2 days and then in starvation medium for 1 h. (c) Starvation medium containing OA for 8 h. **c** Western blotting of the total cell lysate and the LD fraction. MEFs overexpressing CCTβ3 were cultured in regular medium containing OA for 8 h (OA) or in starvation medium containing OA for 8 h (EBSS). Twenty micrograms (total cell) and one microgram (LDs) of proteins were loaded. **d**, **e** The effect of combined DGAT1 and ACAT inhibition on the autophagic flux and the isolation membrane/autophagosome. Cells were cultured for 8 h in starvation medium, with or without a mixture of 20 μM T863 and 1 μM avasimibe for the last 2 h. **d** Autophagic flux. A representative result of three independent experiments. **e** Immunolabeling of endogenous LC3. Bar, 10 μm. The total area of LC3 puncta in a cell was compared. The center line indicates the median, box boundaries indicate the 25th and 75th percentiles, whiskers are Tukey-type, and the average is marked as +. Pooled data obtained in three independent experiments (*n* = 90); ****p < 0.0001 (two-tailed Mann–Whitney test). **f** The effect of CQ in MEFs cultured in starvation medium for either 2 or 8 h. CQ (20 μM) was added to the medium for the last 15, 30, 45, or 60 min and the increase of LC3-II was quantified. A representative result of three independent experiments. Source data are provided as a Source data file.

In MEFs that were starved for 8 h, CCTβ3-mCherry was recruited to LDs (Fig. 5a, Supplementary Fig. 5A). The recruitment of CCTβ3 to LDs was also observed when MEFs were incubated in EBSS containing 62 μM choline or treated with 0.25 μM Torin1 in regular medium (Supplementary Fig. 5B). Endogenous CCTβ also showed distribution in LDs (Supplementary Fig. 5C). In contrast, CCTα-mCherry remained diffusely distributed in the nucleus (Fig. 5a); CCTβ2-mCherry was occasionally observed as dots in late endosome/lysosomes, but was not distributed to LDs (Fig. 5a, Supplementary Fig. 5D).

LDs can be induced to form by adding oleic acid (OA) to regular medium, but those OA-induced LDs did not recruit CCTβ3-mCherry (Fig. 5b). Even when MEFs harboring OA-induced LDs were starved for 1 h, CCTβ3-mCherry was not recruited to the LDs (Fig. 5b). In contrast, when MEFs were cultured for 8 h in EBSS containing OA, CCTβ3-mCherry showed more intense accumulation to LDs than in MEFs cultured in EBSS alone (Fig. 5b). Under this condition, the autophagic flux also increased (Supplementary Fig. 5E). The CCTβ3 recruitment to LDs in long-starved cells, but not to the OA-induced LDs in regular medium, was verified by western blotting of isolated LDs (Fig. 5c). These results indicated that only LDs generated in prolonged starvation recruit CCTβ3.

LDs in long-starved cells are generated from autophagy-derived fatty acids in a diacylglycerol acyltransferase 1 (DGAT1)-dependent manner^17,18^. We found that LDs in long-starved MEFs showed a higher cholesterol ester-to-triglyceride (TG) ratio than did LDs that were induced by OA in regular medium (Supplementary Fig. 5F). Consistent with this lipid ester composition, a combination of DGAT1 and ACAT inhibitors, T863^27^ and avasimibe^28^, is more effective than T863 alone in suppressing LD formation in prolonged starvation (Supplementary Fig. 5G). Simultaneous inhibition of DGAT1 and ACAT for 2 h decreases the autophagic flux and the total isolation membrane/autophagosome significantly (Figs. 5d, e). These results indicated that recruitment of CCTβ3 to LDs made from autophagic digests is important for the continuation of autophagy in prolonged starvation.

Interestingly, by inhibiting adipose triglyceride lipase (ATGL) with Atglistatin, the autophagic flux in MEFs cultured in EBSS for 8 h was decreased (Supplementary Fig. 5H). This result suggests that the hydrolysis of TG stored in LDs is also important for sustaining autophagy in prolonged starvation.

Recognition of the importance of LDs for autophagy led us to reconsider the use of lysosomal inhibitors to measure autophagic flux in the LC3 turnover assay^29,30^. That is, lysosomal inhibition decreases LD formation from autophagic digests^17^ (Supplementary Fig. 5I), and this might downregulate autophagy by suppressing PC synthesis. Upon chloroquine (CQ) treatment, LC3-II increased linearly in MEFs that were starved for 2 h, whereas, in cells starved for 8 h, LC3-II reached a plateau at 45 min and did not increase further (Fig. 5f). This result confirmed the importance of LDs in sustaining autophagy, and indicated that the autophagic flux in long-starved cells may be underestimated in the LC3 turnover assay if lysosomes are inhibited for too long. Based on the above result, we used 30 min of CQ treatment to examine the autophagic flux throughout this study.

### Autophagic membranes form around autophagy-derived LDs

**Fig. 6:**
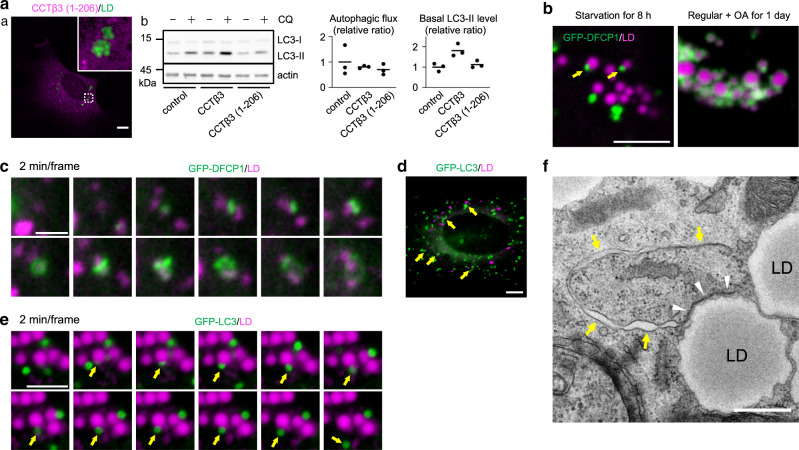
Autophagic membranes and LDs. **a** Distribution and effects of CCTβ3(1-206)-mCherry expression in MEFs starved for 8 h. (a) Distribution of CCTβ3(1-206)-mCherry (magenta) and LDs (green). Bar, 10 μm. (b) Western blotting of LC3. MEFs overexpressing CCTβ3 or CCTβ3(1-206) were compared with untransfected MEFs. A representative result of three independent experiments. **b** Distribution of GFP-DFCP1 (green) and LDs (magenta) in MEFs starved for 8 h (left) and in MEFs cultured in OA-containing regular medium for 1 day (right). GFP-DFCP1 puncta in starved MEFs are marked by arrows. Bar, 5 μm. **c** Expansion of the omegasome (green, GFP-DFCP1) around LDs (magenta) in MEFs starved for 8 h. Selected images from Supplementary Movie 1. Bar, 2 μm. **d** Distribution of GFP-LC3 (green) and LDs (magenta) in MEFs starved for 8 h. Bar, 10 μm. **e** Emergence of isolation membranes/autophagosomes (green, GFP-LC3; arrows) around LDs (magenta) in MEFs starved for 36 h. Selected images from Supplementary Movie 2. Bar, 5 μm. **f** EM of MEFs overexpressing CCTβ3 starved for 8 h. The isolation membrane (arrows) adheres to the LD surface (arrowheads). Bar, 0.5 μm. Source data are provided as a Source data file.

The above results indicated that CCTβ3 activation in autophagy-derived LDs promotes continuous autophagosome formation. In contrast, overexpression of a constitutive-active CCTβ3 mutant, CCTβ3(1-206)^26^, which is not recruited to LDs, upregulated PC synthesis similarly but did not increase either the basal LC3-II level or the autophagic flux (Fig. 6a, Supplementary Fig. 6A). This result suggested that CCTβ3 may need to be activated in LDs to facilitate autophagic membrane formation. We thus hypothesized that PC synthesis and the formation of autophagic membranes in long-starved MEFs may occur around LDs.

Consistent with this idea, GFP-DFCP1 puncta were distributed close to LDs in MEFs starved for 8 h (Fig. 6b). This distribution was different from that in MEFs in OA-containing regular medium, in which GFP-DFCP1 encircled LDs (Fig. 6b)^31,32^. Moreover, live imaging showed that the GFP-DFCP1 signal in starved MEFs originates and expands from around LDs (Fig. 6c, Supplementary Movie 1). Isolation membranes/autophagosomes marked by GFP-LC3 were also frequently observed near LDs (Fig. 6d), and live imaging showed their emergence from around LDs (Fig. 6e, Supplementary Movie 2). The outgrowth of isolation membranes/autophagosomes from around LDs was observed more frequently in MEFs overexpressing CCTβ3 (Supplementary Fig. 6B, Supplementary Movie 3). De novo-synthesized PC was labeled intensely on the LD rim, which may correspond to emanating autophagic membranes (Supplementary Fig. 6C).

Consistent with the result of fluorescence microscopy, EM showed that isolation membranes, identified as a thin cisterna bound with electron-dense membranes, were frequently associated with LDs in MEFs that were starved for 8 h (Fig. 6f, Supplementary Fig. 6D). This contrasted with the distribution of isolation membranes adjacent to the ER, or the cradle membrane, in MEFs that were starved for 1 h (Supplementary Fig. 6E). Neither LC3 nor DFCP1 was enriched in LDs isolated from MEFs starved for 8 h, suggesting that LDs and autophagic membranes do not bind tightly (Fig. 5c). Nevertheless, these results indicated that autophagic membranes in long-starved cells preferentially form in the vicinity of autophagy-derived LDs.

### CCTβ3 sustains prolonged autophagy and survival of cancer cells

Cancer cells may utilize autophagy for survival in host tissues^33^. Considering that unfavorable conditions for cancer cells may last for a long period of time, e.g., hypoxia resulting from insufficient blood supply, the CCTβ3-dependent mechanism may be involved in sustaining prolonged autophagy and thereby support their survival. To test this idea, U2OS, a human osteosarcoma cell line, was examined.

**Fig. 7:**
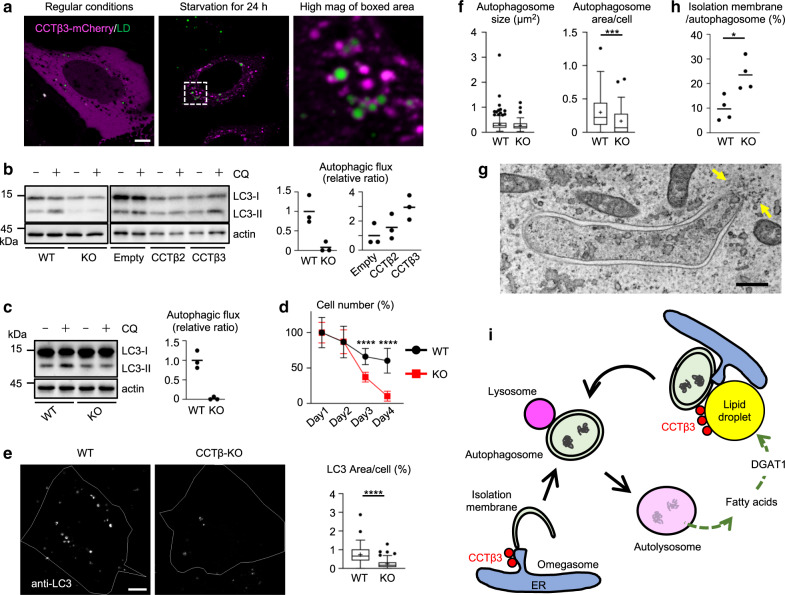
CCTβ3 in U2OS. **a** Distribution of CCTβ3-mCherry (magenta) and LDs (green). Bar, 10 μm. **b** The autophagic flux after starvation for 24 h. Wild-type U2OS and CCTβ-null U2OS (left panel), and CCTβ-null U2OS transfected with an empty vector, CCTβ2 cDNA, or CCTβ3 cDNA (right panel) were compared. A representative result of three independent experiments. **c** The autophagic flux after 2 days of hypoxia (0.1% O_2_) in regular medium. A representative result of two independent experiments. **d** Cell survival in prolonged starvation. Cells were plated on day 0 and switched to starvation medium on day 1. The number of living cells on day 1 was set as 100%. Pooled data obtained in two independent experiments. Mean ± s.d. of 12 samples. ****p < 0.0001 (unpaired two-tailed *t*-test). **e** Endogenous LC3 in wild-type and CCTβ-null U2OS cultured for 24 h in starvation medium. Bar, 10 μm. Pooled data obtained in three independent experiments (*n* = 90); ****p < 0.0001 (two-tailed Mann–Whitney test). **f** Quantification of autophagosomes by EM. Wild-type and CCTβ-null U2OS cultured for 24 h in starvation medium. The average autophagosome size (left) and the total area of autophagosomes in the cytoplasm (right). Pooled data from four independent experiments. ***p = 0.0010 (two-tailed Mann–Whitney test). The number of cells examined: 45 (wild-type) and 41 (CCTβ-null). The number of autophagosomes examined: 249 (wild-type) and 83 (CCTβ-null). **g** Elongated isolation membrane in CCTβ-null U2OS starved for 24 h. Bar, 0.2 μm. IMAT membranes (arrows). See Supplementary Fig. 7G for additional micrographs. **h** The numerical ratio of isolation membranes to autophagosomes measured by EM. Wild-type and CCTβ-null U2OS cultured for 24 h in starvation medium. Four independent experiments. *p = 0.0286 (two-tailed Mann–Whitney test). The number of isolation membranes observed: 24 (wild-type) and 21 (CCTβ-null). **i** CCTβ3 in autophagy. In cells starved for a short time, CCTβ3 is activated near autophagic membranes. After prolonged starvation, CCTβ3 is recruited to LDs generated from autophagic digests, and the resultant activation of PC synthesis facilitates autophagic membrane formation near LDs, thereby sustaining autophagy. In box plots, the center line indicates the median, box boundaries indicate the 25th and 75th percentiles, whiskers are Tukey-type, and the average is marked as +. Source data are provided as a Source data file.

In U2OS cells that were starved for 24 h, LDs increased and CCTβ3 was recruited to LDs, as observed in MEFs (Fig. 7a). GFP-DFCP1 in starved U2OS also showed distribution near LDs (Supplementary Fig. 7A). In CCTβ-null U2OS generated by the CRISPR/Cas method (Supplementary Fig. 7B), autophagy induced by prolonged starvation or hypoxia (0.1% O_2_) was significantly suppressed (Fig. 7b, c), and this autophagic defect was rescued by re-expression of human CCTβ3 (NP_001156736.1), but only less efficiently by that of human CCTβ2 (NP_004836.2) (Fig. 7b). Moreover, when kept in starvation medium, the number of CCTβ-null U2OS decreased much earlier than the control U2OS did (Fig. 7d). Re-expression of CCTβ3 in CCTβ-null U2OS increased the number of surviving cells after starvation, whereas that of CCTβ2 or CCTβ3 mutants defective in the catalytic activity, CCTβ3 (K104G), or membrane binding, CCTβ3 (1-218), did not (Supplementary Fig. 7C). Decreases in the autophagic flux and cell survival were also observed by knockdown of either CEPT1 or CHPT1, which catalyze the final step of the Kennedy pathway, verifying that PC synthesis is required for prolonged autophagy (Supplementary Fig. 7D).

CCTβ3 was also recruited to LDs in Huh7, a hepatocellular carcinoma cell line, after prolonged starvation (Supplementary Fig. 7E). In CCTβ-null Huh7, the PC synthetic activity by the Kennedy pathway was suppressed, but the autophagic flux did not decrease (Supplementary Fig. 7E), suggesting that the phosphatidylethanolamine N-methyltransferase pathway suffices for sustaining autophagy in Huh7.

CCTβ depletion did not affect the size of individual autophagosomes, but caused a significant decrease in the total autophagosome area in a cell; this was evident both by immunofluorescence microscopy of endogenous LC3 (Fig. 7e) and by EM (Fig. 7f, Supplementary Fig. 7B). GFP-LC3 in CCTβ-null U2OS frequently showed elongated shapes in live imaging (Supplementary Movies 4, 5). Correspondingly, highly elongated isolation membranes associated with tubular/vesicular structures (IMAT)^34^ were observed by EM (Fig. 7g, Supplementary Fig. 7G). Moreover, the ratio of isolation membranes to autophagosomes was significantly higher in CCTβ-null U2OS than in control U2OS (Fig. 7h). These results suggested that the absence of CCTβ might retard the process of autophagosomal maturation in U2OS.

This result indicated that CCTβ3 activated on LDs that are generated from autophagic digests is also important for sustaining prolonged autophagy in U2OS (Fig. 7i).

## Discussion

The ER and its subdomain are thought to be the major platform of autophagosome formation^6,7,9,34^, but the source of phospholipids composing the autophagosomal membrane remains unclear^14^. In the present study, we found that autophagosomes in mammalian cells incorporate newly synthesized PC at a higher density than the ER membrane. This result is in line with a recent report on budding yeast, which showed that fatty acids activated by acyl-CoA synthetase Faa1 are channeled to de novo phospholipid synthesis and promote isolation membrane expansion^35^. Furthermore, we identified CCTβ3 as a critical enzyme for activating PC synthesis in starvation-induced autophagy, and showed its increased importance as starvation is prolonged. Among three CCT isoforms that are expressed in rodents, CCTα is predominant, ubiquitously expressed and essential for survival^36^, and CCTβ2 functions in neurons and gonads^37^, whereas the significance of CCTβ3 was not known despite its ubiquitous, albeit low, expression in adult mouse tissues^23^. The present result clarified the physiological function of CCTβ3. This result differs from that of a recent study that concluded CCTα as the critical CCT isoform in autophagosome formation^38^. We argue that the prolonged loading of propargylcholine used for PC labeling in the study^38^ (i.e., 6 vs. 1 h pulse loading in the present study) is not appropriate for visualizing de novo-synthesized PC because all cellular membranes including cargos engulfed by autophagosomes are intensely labeled by the protocol (see Fig. 3 of ref. ^38^).

De novo PC synthesis via the Kennedy pathway requires choline, but it is not contained in the starvation medium or synthesized endogenously in most cell types lacking phosphatidylethanolamine N-methyltransferase^39^. Thus, choline is likely to be derived from PC, lysophosphatidylcholine, and sphingomyelin, which are digested in the lysosome or by cytoplasmic phospholipases. In this context, it is notable that phospholipase D1 promotes autophagy^40–43^ (although an opposite result was also reported^44^). The effect of phospholipase D1 on autophagy has been ascribed to the modulation of the phosphatidic acid level, but we speculate that the generation of choline, which is used for PC synthesis, is also an important function of this enzyme reaction.

In prolonged autophagy, CCTβ3 is activated on LDs, so that its product, CDP-choline, must be concentrated around those LDs. On the other hand, diacylglycerol, which reacts with CDP-choline to produce PC, may be derived from the hydrolysis of TG stored in LDs^45,46^. Thus, it is reasonable to think that the final PC synthesis occurs most efficiently in the vicinity of LDs. This localization of PC synthesis around LDs appears to be critical for autophagosome formation, because the constitutive-active CCTβ3 mutant that is distributed diffusely in the cytoplasm increases PC synthesis to a similar extent as wild-type CCTβ3 does but fails to upregulate autophagy.

CEPT1 and phosphatidylinositol synthase (PIS) are enriched in a Rab10-positive ER domain^47^. This ER domain is probably similar to another PIS-rich domain, which recruits the autophagy-initiation complex in starvation and is likely to harbor CEPT1, CHPT1, phosphatidylserine synthase l, and phospholipase D1A^48^. The interrelationship between these CEPT1-PIS-rich domains and the phosphatidylinositol 3-phosphate (PI3P)-rich omegasome is not clear, but the autophagy-initiation complex is translocated from the CEPT1-PIS-rich domain to the ATG9A-positive membrane in a PI3P-dependent manner^48^, and the ATG9A-positive membrane shows transient colocalization with the omegasome^49,50^. In conjunction with the LD–omegasome association found in the present study, it is inferred that the CEPT1-PIS-rich ER domain located around LDs is where PC and PI are synthesized for autophagosome biogenesis.

We found that LDs generated from autophagic digests in long-starved cells show different characteristics from those in nutrient-rich regular medium: that is, CCTβ3 is recruited to autophagy-derived LDs but not to others, and DFCP1 encircles LDs in the nutrient-rich condition^31,32^, but not those in long-starved cells. Frequent emergence of isolation membranes/autophagosomes and omegasomes around LDs is probably based on these unique properties of autophagy-derived LDs. LDs have already been shown to play various roles in autophagy—a substrate of lipophagy^51^, a source of lipids for autophagosome formation^45,46,52^, a product made from autophagic digests^17,18^, and the maintenance of ER homeostasis^53^, among others^54^. Besides these roles, the present results indicated that LDs serve as the platform for PC synthesis activation and that this role becomes more important as the duration of autophagy increases.

The function of LDs in long-term autophagy may not be fulfilled if LDs are consumed for mitochondrial β-oxidation more rapidly than they are generated. However, LDs in starved MEFs continue to increase for the first 12 h or so and maintain their peak level thereafter^17^. This is probably because the lipid-driven mitochondrial oxygen consumption rate reaches a plateau early in starvation^18^. Thus, LDs formed as a result of autophagy can work continuously as a platform to generate new autophagosomes, thereby sustaining autophagy for a long duration.

Most experiments on autophagy used cultured cells and examined autophagy for a relatively short period of time, such as 1–3 h, but autophagy in vivo may last longer. One prominent example of prolonged autophagy may be found in solid tumors, which are exposed to hypoxic and nutrient-deficient conditions resulting from insufficient blood supply^55,56^. Whether the LD-based activation of CCTβ3 occurs in cancer cells in vivo remains to be addressed, but it is intriguing that cancer cells form many LDs in hypoxia^57^. Moreover, cancer cells are highly active in choline metabolism and express PC synthesis enzymes abundantly^58^. It is reasonable to think that PC synthesis that is driven by the LD-based CCTβ3 activation may play an important role for cancer survival in vivo.

Inhibition of autophagy is thought to be a promising avenue for cancer therapy, but clinical studies using CQ and hydroxychloroquine have shown mixed results^59^. An obvious problem with using autophagy inhibitors is that they are not selective to cancer cells and may affect healthy tissues, in which autophagy is important for maintaining homeostasis. Moreover, lysosome inhibitors such as CQ and hydroxychloroquine have effects other than autophagy suppression. We expect that CCTβ3 may be an excellent drug target for cancer therapy because it is dispensable for short-term autophagy in normal cells. Studies in this direction are certainly warranted.

## Methods

### Cell culture and reagents

MEFs without or with the expression of GFP-ULK1, GFP-WIPI1, GFP-DFCP1, or GFP-LC3^20^ were kindly provided by Dr. Noboru Mizushima (University of Tokyo). U2OS and Huh7 cells were kindly donated by Dr. Hidemasa Goto (Mie University) and Dr. Eija Jokitalo (University of Helsinki), respectively. MEFs and U2OS were cultured in Dulbecco’s modified Eagle medium supplemented with 10% FBS and antibiotics. For Huh7, Eagle’s minimum essential medium supplemented with 10% FBS and antibiotics were used. The cells were maintained at 37 °C in a humidified atmosphere of 95% air and 5% CO_2_. The total choline concentration in the medium was measured using LabAssay^TM^ Phopholipid (296-63801, Fujifilm Wako Pure Chemical). Autophagy was induced by culturing cells in a serum-free EBSS (6.8 mg ml^–1^ NaCl, 0.4 mg ml^–1^ KCl, 0.16 mg ml^–1^ NaH_2_PO_4_•2H_2_O, 0.26 mg ml^–1^ CaCl_2_•2H_2_O, 0.21 mg ml^–1^ MgSO_4_•7H_2_O, 2.2 mg ml^–1^ NaHCO_3_, 1 mg ml^–1^ glucose). OA was conjugated with fatty acid-free BSA (Fujifilm Wako Pure Chemical) at a 6:1 molar ratio before being added to the medium at a final concentration of 0.4 mM^60^. Hypoxic conditions were produced using the BIONIX-3 hypoxic culture kit (Sugiyama-gen). T863 (SML0539-5MG, Sigma), avasimibe (A794680, Toronto Research Chemicals), and Atglistatin (15284, Cayman Chemical) were used for the inhibition of DGAT1, ACAT, and ATGL, respectively.

### Antibodies

A rabbit antibody binding to the common C-terminal portion of mouse and human CCTβ2 and CCTβ3 were generated using a synthetic peptide (SASISSMSEGDEDEK) and affinity-purified. Another rabbit antibody binding to both CCTβ2 and CCTβ3^61^ was kindly provided by Dr. Suzanne Jackowski (St. Jude Children’s Research Hospital). Rabbit anti-GFP and anti-DsRed antibodies were kindly provided by Dr. Masahiko Watanabe (Hokkaido University). Rabbit antibodies to Tom20 (sc-11415, Santa Cruz), cytochrome P450 (BV-3084-3, MBL), LC3 (NB100-2220, Novus), actin (A2066, Sigma-Aldrich), perilipin-2 (PA1-16972, Thermo Fisher), DFCP1 (85156, Cell Signaling), and CEPT1 (AP10372a, Abgent), mouse antibodies to EEA1 (610457, BD Biosciences), LC3 (M152-3, MBL), and CHPT1 (sc-515577, Santa Cruz), and rat anti-Lamp1 antibody (1D4B, Developmental Studies Hybridoma Bank) were obtained from the respective suppliers. Secondary antibodies conjugated to fluorochromes (Thermo Fisher and Jackson ImmunoResearch Lab) and protein A-conjugated 10 nm colloidal gold particle (PAG10, Utrecht University Medical Center) were also purchased. The primary antibodies were used at 0.1–1 μg ml^–1^ for western blotting and at 10 μg ml^–1^ for immunofluorescence and immunoelectron microscopy. Cy3- and Alexa488-tagged secondary antibodies and PAG were used in 1/3,000, 1/200, and 1/30 dilution of the supplied solution, respectively.

### Plasmids

pEGFP-Sec23A (Addgene plasmid # 66609)^62^ was a gift from Dr. David Stephens. Plasmids encoding mouse and human CCTα, β2, β3, and β3 mutants were cloned by PCR using cDNA of mouse and human cells as templates and tagged with mCherry at the C terminus. Primer sequences for PCR are provided in Supplementary Table 1. Plasmids were transfected to MEFs and U2OS using Lipofectamine 2000 or Lipofectamine 3000 (Thermo Fisher).

### siRNA and shRNA

The siRNAs used in the present study were mouse CCTβ #2, 5′-CUUAAGUGGGUUUAAACUTT-3′ and mouse CCTβ #3, 5′-GCUCAUUAGGCAAAUUUUTT-3′ (synthesized by Japan Bio Service). The siRNAs were transfected using Lipofectamine RNAiMAX (Thermo Fisher). For human CEPT1 and CHPT1, stable cell lines harboring shRNAs (TR313975D and TR305398C, Origene) were generated as described below using retroviral vectors.

### Retroviral production and generation of stable cell lines by infection

PLAT-E cells^63^ and pMXs-IRES-Puro vector^64^ were kindly provided by Dr. Toshio Kitamura (University of Tokyo). PLAT-E cells were transfected with pMXs-IRES-Puro vector carrying an appropriate insert using polyethylenimine MAX^65^. To produce retroviruses transducing U2OS cells, pcDNA3.1-Vesicular Stomatitis Virus G protein was co-transfected with pMXs-IRES-Puro. Two days after transfection, the culture medium was collected, filtered, and applied to cells in the presence of 5 μg ml^–1^ polybrene. Cells stably expressing transfected cDNAs and shRNAs were obtained by selection in medium containing 2 μg ml^–1^ puromycin.

### Gene knockout by CRISPR-Cas9 system

CCTβ in U2OS and Huh7 was disrupted according to the standard protocol^66^. Nucleotide sequences (467–486 for U2OS and 429–448 for Huh7) in PCYT1B mRNA (Accession No. NM_004845.5) that are commonly used for CCTβ2 and CCTβ3 were selected using web-based prediction software (Crispr direct; http://crispr.dbcls.jp)^67^ and transcribed with a MEGAshortscript T7 Transcription Kit (Thermo Fisher). Cells were co-transfected with the in vitro-transcribed guide RNA and GeneArt CRISPR Nuclease mRNA (Thermo Fisher) using Lipofectamine Messenger MAX (Thermo Fisher) and examined for genome digestion by GeneArt Genomic Cleavage Detection Kit (Thermo Fisher) 2 days after transfection. Clones obtained by single-cell cloning were checked by DNA sequencing to confirm genome editing and western blotting.

### Cell viability assay

U2OS (3–5 × 10^5^ cells/well) was propagated to a six-well plate and the culture medium was changed to EBSS 1 day later. For the rescue experiment using CCTβ-null U2OS, cDNAs were introduced by reverse transfection 1 day before the EBSS switching. After incubation in EBSS for 1–3 days, cells were detached from the substrate by treatment with trypsin and EDTA and briefly incubated in 0.2% trypan blue. The number of unstained live cells was counted using a hemocytometer or TC20 Automated Cell Counter (Bio-Rad).

### LD fractionation

Cells rinsed with ice-cold PBS containing 5 mM EDTA were disrupted in homogenizing buffer (25 mM Tris-HCl, 100 mM KCl, 1 mM EDTA, 5 mM EGTA, protease inhibitor cocktail [Nacalai], pH 7.4) by passing through a 26 G needle more than 10 times. The post-nuclear supernatant was mixed with an equal volume of 1.08 M sucrose in homogenizing buffer, overlaid with 0.27 M sucrose, 0.135 M sucrose, and top solution (25 mM Tris-HCl, 1 mM EDTA, 1 mM EGTA, pH 7.4), and ultracentrifuged for 90 min at 45,000 rpm using a TLX55 rotor in an Optima TLX ultracentrifuge (Beckman)^68^. LDs recovered from the top layer were precipitated by adding 10% trichloroacetic acid.

### Western blotting

Total cells and LDs were dissolved in SDS sample buffer (2% SDS, 10% glycerol, 60 mM Tris-HCl, pH 6.8), and protein concentration was determined by BCA assay (Pierce). Two to twenty micrograms (for total cell) or one microgram (for LDs) of protein was electrophoresed in SDS–polyacrylamide gel and transferred to nitrocellulose membranes. After incubation with appropriate antibodies, the reaction obtained with Super Signal West Dura Substrate (34076, Thermo Fisher) was visualized using a Fusion Solo S instrument (Vilber Lourmat) and analyzed by Fusion-Capt Advance Software version 16.15.

### Assay of autophagic flux

Cells cultured in various conditions were treated without or with 20 μM CQ (C6628-25G, Sigma) for the last 30 min and examined for LC3 by western blotting. The autophagic flux was estimated by the CQ-induced increase of LC3-II normalized by the basal LC3-II level in cells without the CQ treatment.

### Assay of PC synthetic activity

Cells were cultured with 1 μCi ml^–1 3^H-choline (NET109250UC, Perkin Elmer) for 30 min and incubated with hexane/isopropanol (9:1) for 30 min to selectively extract PC^69^. The ^3^H-choline radioactivity was measured using a liquid scintillation counter (Aloka) and normalized to the protein concentration.

### Lipid extraction and thin layer chromatography

The LD fraction was mixed with 20 volume of chloroform–methanol mixture (2:1) and subjected to a low-speed centrifugation. The supernatant was mixed with 0.9% sodium chloride solution, centrifuged, and the lower organic phase layer was used for the analysis^70^. For TLC, the sample was dried, dissolved in chloroform/methanol (1/1), and separated on a HPTLC Silica gel 60 plate (1.05631.0001, Merck) by hexane/diethylether/acetic acid (80/20/1). Lipid spots on the plate were visualized by incubation in 3% copper acetate in 85% phosphoric acid and heating at 180 °C. The density of spots was measured in ImageJ, and the amounts of CE and TG in LD samples were estimated by standard curves prepared by the results of standard samples.

### PC labeling by click chemistry

Propargylcholine bromide was synthesized from propargyl-bromide and dimethyl-ethanolamine^19^. Cells were cultured with 0.25 mM or 2.5 μM propargylcholine, fixed for 15 min with 4% formaldehyde in 0.1 M phosphate buffer, and incubated for 20 min in reaction solution containing 10 nM Cy3-azide (BCFA-080-1, baseclick), 1 mM CuSO_4_, 10 μM Tris[(1-benzyl-1H-1,2,3-triazol-4-yl)methyl]amine (35434-21, Nacalai Tesque), 2.5 mM ascorbic acid, 2% cold water fish skin gelatin (G7041, Sigma), and 0.1 M Tris-HCl (pH 7.5).

### Fluorescence microscopy and data analysis

For live imaging, cells cultured in a glass-bottom dish (3911-035, IWAKI) were kept at 37 °C, and images were taken every 30 s. For immunofluorescence microscopy, cells were fixed with 4% formaldehyde in 0.1 M phosphate buffer for 15 min, permeabilized with 0.01% digitonin in PBS for 30 min, and labeled with antibodies. BODIPY493/503 and LipidTox Red (Thermo Fisher) were used to stain LDs.

Images of random areas were captured by an Axiovert 200 M microscope equipped with Apotome2 (Zeiss) using an Apochromat 63× lens with a 1.40 numerical aperture. The color, brightness, and contrast of the presented images were adjusted using Adobe Photoshop CC.

To quantify the colocalization of two different labels, images in red and green channels were processed by ImageJ for background subtraction using the rolling ball method without smoothing. The radius size was set at 10 (PC, CCTβ3, and EEA1), 5 (WIPI1 and DFCP1), or 2.5 (ULK1), to best reflect the punctate pattern of respective labels. To quantify colocalization, the ratio of Atg protein pixels overlapping with PC or CCTβ3 puncta was used.

### Electron microscopy of ultrathin sections

For conventional ultrathin section electron microscopy, cells cultured on coverslips were fixed with a mixture of 2% formaldehyde and 2.5% glutaraldehyde in 0.1 M HEPES buffer (pH 7.4) for more than 2 h at room temperature, and post-fixed with a mixture of 1% osmium tetroxide and 0.1% potassium ferrocyanide in 0.1 M sodium cacodylate buffer (pH 7.4)^71^. For delineation of isolation membranes and IMATs, cells were fixed for 40 min at 4 °C with a glutaraldehyde–osmium tetroxide mixture using a slight modification of the published methods^34,72^. The fixative was prepared by mixing one volume of solution A (2.5% glutaraldehyde, 0.1 M sodium cacodylate buffer [pH 7.4]) and two volumes of solution B (1% osmium tetroxide, 3 mg ml^–1^ potassium ferrocyanide, 0.1 M sodium cacodylate buffer [pH 7.4]) immediately before use. All the samples were dehydrated, and embedded in epoxy resin. Ultrathin sections were observed using a JEOL JEM1011 electron microscope operated at 100 kV. Digital images were captured using a CCD camera (Gatan).

### Electron microscopy of freeze-fracture replicas

MEFs expressing GFP-LC3 were cultured in EBSS containing 0.25 mM propargylcholine for 1 h and quick-frozen using an HPM 010 high-pressure freezing machine (Leica). The frozen cells were freeze-fractured in a Balzers BAF 400 apparatus at −105 to −115 °C, and replicas were made by electron-beam evaporation^22^. Thawed replicas were treated overnight with 2.5% SDS in PBS at 60 °C and subjected to label PC or GFP-LC3. For PC labeling, replicas were incubated for 30 min in reaction solution containing 5 μM biotin-azide (baseclick), 1 mM CuSO_4_, 100 μM Tris[(1-benzyl-1H-1,2,3-triazol-4-yl)methyl]amine, 2.5 mM ascorbic acid, 2% cold water fish skin gelatin, and 0.1 M Tris-HCl (pH 7.5), followed by incubation with anti-biotin antibody and then by colloidal gold (10 nm)-conjugated donkey anti-mouse IgG antibody^21^. For labeling of GFP-LC3, replicas were incubated with rabbit anti-GFP antibody followed by colloidal gold (10 nm)-conjugated protein A. Labeled replicas were examined by electron microscope.

### Statistical methods and reproducibility

Statistical differences between samples were examined by two-tailed Mann–Whitney test or unpaired two-tailed *t*-test using Prism 8 (GraphPad), except for Fig. 2c. The analysis in Fig. 2c was done by the two-tailed Wilcoxon signed-rank test using the statistical language R. When statistical significance exists, the *p* values above 0.0001 are shown in exact numbers and those below 0.0001 are indicated by four asterisks (****). In box plots, the center line indicates the median, box boundaries indicate the 25th and 75th percentiles, whiskers are Tukey-type, and the average is marked as +. No statistical method was used to predetermine sample size. Unless otherwise stated, the experiments were performed at least three times and yielded similar results. All representative images reflect a minimum of three biological replicates.

### Reporting summary

Further information on experimental design is available in the Nature Research Reporting Summary linked to this paper.

## Supplementary information

Supplementary Information

Peer Review File

Supplementary Movie 1

Supplementary Movie 2

Supplementary Movie 3

Supplementary Movie 4

Supplementary Movie 5

Reporting Summary

## Data Availability

The authors declare that all data supporting the findings of this study are available within the article and its supplementary information files or from the corresponding author upon reasonable request. Source data are provided with this paper. Annotations for mouse CCTβ isoforms are found at the NCBI database as CCTβ2 [https://www.ncbi.nlm.nih.gov/protein/NP_997593.1] CCTβ3 [https://www.ncbi.nlm.nih.gov/protein/NP_808214.1].

## Source data

Source Data
