# Supplementary material for: Long-term autophagy is sustained by activation of CCTβ3 on lipid droplets
Source: Nat Commun. 2020 Sep 8;11:4480. doi: 10.1038/s41467-020-18153-w (PMC7479109; doi:10.1038/s41467-020-18153-w)
Supplement: Supplementary file 1 — Supplementary Information [file 41467_2020_18153_MOESM1_ESM.pdf]

Supplementary Information

Ogasawara et al.

Long-term autophagy is sustained by activation of CCTβ3 on lipid droplets

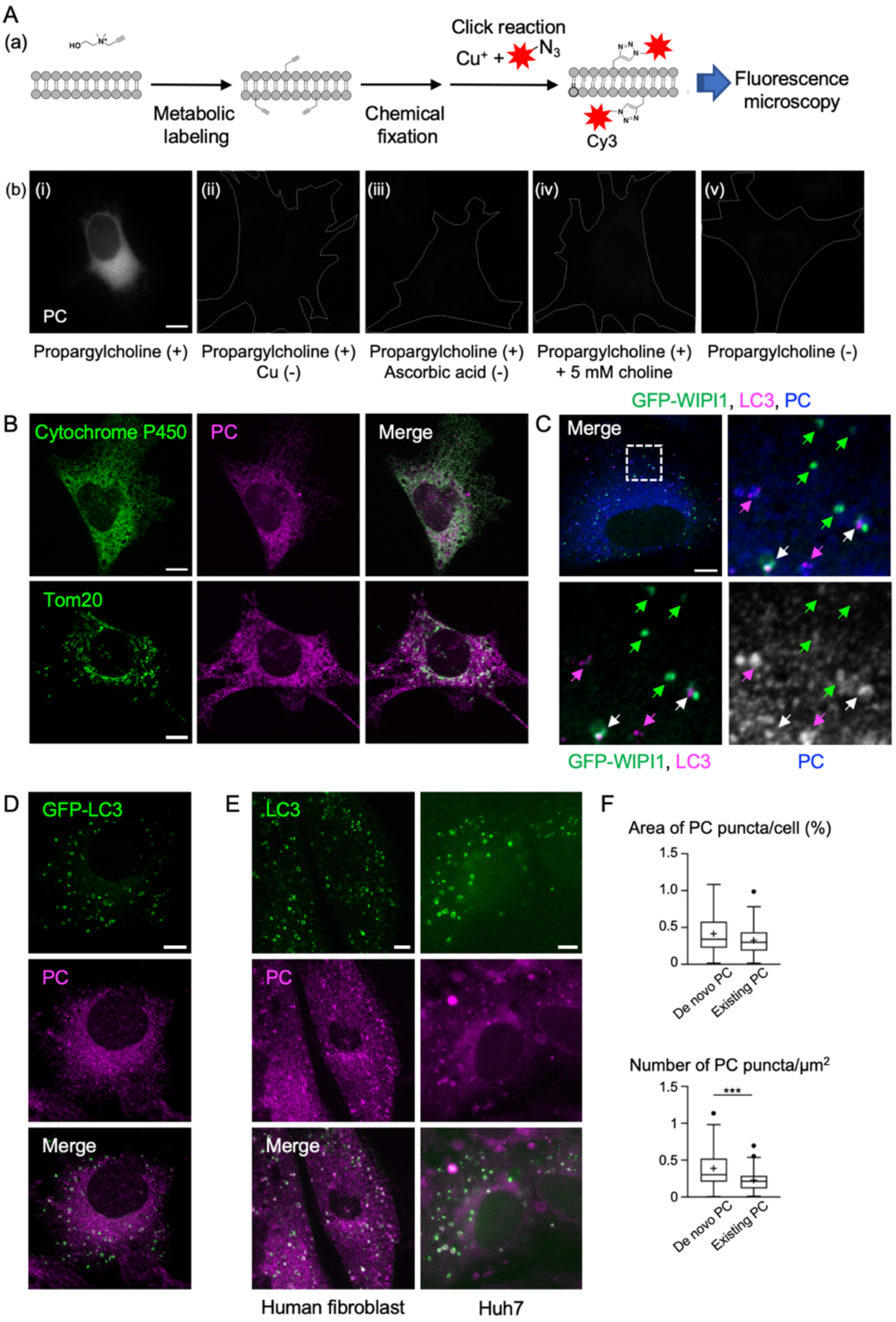
A
(a)
Click reaction
Cu+ + N3
Metabolic labeling
Chemical fixation
Cy3
Fluorescence microscopy
(b) (i) (ii) (iii) (iv) (v)
PC
Propargylcholine (+)
Propargylcholine (+) Cu (-)
Propargylcholine (+) Ascorbic acid (-)
Propargylcholine (+) + 5 mM choline
Propargylcholine (-)
B
Cytochrome P450
PC
Merge
Tom20
C
GFP-WIPI1, LC3, PC
Merge
GFP-WIPI1, LC3
PC
D
GFP-LC3
PC
Merge
E
LC3
PC
Merge
Human fibroblast
Huh7
F
Area of PC puncta/cell (%)
1.5
1.0
0.5
0
De novo PC
Existing PC
Number of PC puncta/μm2
***
1.5
1
0.5
0
De novo PC
Existing PC

Supplementary Fig. 1. Fluorescent labeling of *de novo*-synthesized PC

(A) *De novo*-synthesized PC labeling. (a) The outline of the method. Cells were cultured for 1 h with propargylcholine, a choline analog with an alkyne residue. After fixation, alkyne incorporated to the PC head group was conjugated with Cy3-azide by click reaction and observed by fluorescence microscopy. (b) Control experiments. MEFs cultured for 1 h in regular medium containing 0.25 mM propargylcholine and treated in the full click reaction solution (i), in a solution without copper (ii), or in a solution without ascorbic acid (iii). MEFs cultured with 0.25 mM propargylcholine and 5 mM choline (iv) or without propargylcholine (v), and treated in the full click reaction solution. Micrographs were taken by the same setting. Bar, 10 μm.

(B) PC (magenta) and organelle markers (green). MEFs cultured for 1 h with 0.25 mM propargylcholine in regular medium. ER and mitochondria are shown by immunolabeling of cytochrome P450 and Tom20, respectively. Bars, 10 μm.

(C) GFP-WIPI1 (green), endogenous LC3 (magenta), and PC (blue) in MEFs cultured for 1 h with 0.25 mM propargylcholine in starvation medium. PC puncta colocalizing with GFP-WIPI1 alone (green arrows), LC3 alone (magenta arrows), and both GFP-WIPI1 and LC3 (white arrows) are shown. Bar, 10 μm.

(D) PC labeling with a low propargylcholine concentration. MEFs were cultured for 1 h in starvation medium containing 2.5 μM propargylcholine and treated in the full click reaction solution. PC (magenta), GFP-LC3 (green). Bar, 10 μm.

(E) Distribution of PC (magenta) and endogenous LC3 (green) in human primary fibroblasts and Huh7 cells cultured for 1 h with 0.25 μM Torin1 and 0.25 mM propargylcholine in regular medium. Bars, 10 μm.

(F) Labeling of existing PC. The PC labeling was performed as in Fig. 1D, except that starvation medium was added with 62 μM choline. Bar, 10 μm. The total area and the number of PC puncta in a cell were measured. The center line indicates the median, box boundaries indicate the 25th and 75th percentiles, whiskers are Tukey-type, and the average is marked as +. Pooled data from three independent experiments (n = 60); ****p* = 0.0002 (two-tailed Mann-Whitney test).

Source data are provided as a Source data file.

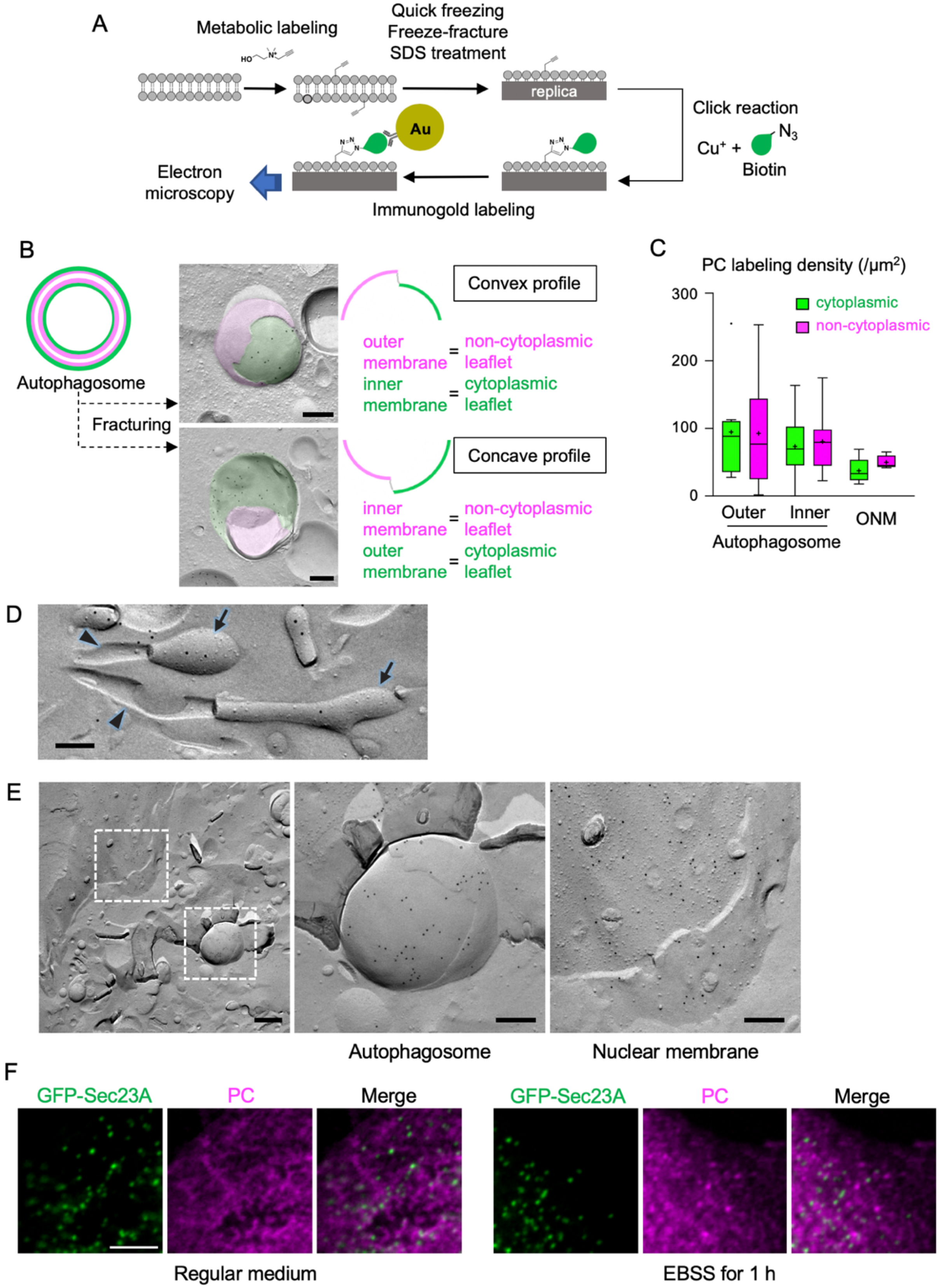
A
Metabolic labeling
Quick freezing
Freeze-fracture
SDS treatment
replica
Click reaction
Cu⁺ +
N3
Biotin
Au
Electron microscopy
Immunogold labeling
B
Autophagosome
Fracturing
Convex profile
outer membrane = non-cytoplasmic leaflet
inner membrane = cytoplasmic leaflet
Concave profile
inner membrane = non-cytoplasmic leaflet
outer membrane = cytoplasmic leaflet
C
PC labeling density (/μm²)
300
200
100
0
cytoplasmic
non-cytoplasmic
Outer
Inner
Autophagosome
ONM
D
E
Autophagosome
Nuclear membrane
F
GFP-Sec23A
PC
Merge
Regular medium
GFP-Sec23A
PC
Merge
EBSS for 1 h

Supplementary Fig. 2. Labeling of *de novo*-synthesized PC in freeze-fracture EM.

(A) The outline of the method. Cells cultured for 1 h with propargylcholine in starvation medium were quick-frozen and freeze-fractured, and freeze-fracture replicas were subjected to the click reaction to conjugate biotin to the alkyne residue in PC. The replica is labeled with anti-biotin antibody and gold-conjugated secondary antibody, and observed by electron microscopy.

(B) Autophagosomes. MEFs expressing GFP-LC3 were cultured for 1 h in starvation medium and freeze-fracture replicas were labeled with anti-GFP antibody. The diagram shows membrane leaflets observed in the convex and concave profiles of the freeze-fracture autophagosome. Bars, 0.2 μm.

(C) PC labeling density in respective membrane leaflets of outer and inner autophagosomal membranes and the outer nuclear membrane (ONM). The center line indicates the median, box boundaries indicate the 25th and 75th percentiles, whiskers are Tukey-type, and the average is marked as +. Pooled data obtained in three independent experiments. The total number of areas measured: 49 (autophagosome) and 14 (ONM). The result combining cytoplasmic and non-cytoplasmic leaflets is shown in Figure 2B.

(D) The PC labeling in the ER is comparable to that in the ONM. The cytoplasmic and non-cytoplasmic leaflets are marked with arrowheads and arrows, respectively. Bar, 0.2 μm.

(E) A representative set of micrographs used to measure PC labeling density in the autophagosome and the ONM of the same cell. MEFs cultured for 1 h in starvation medium. Boxed areas in the left figure are magnified in the middle and right figures. Bars, 0.2 μm.

(F) PC (magenta) and GFP-Sec23 (green). MEFs were cultured for 1 h with 0.25 mM propargylcholine either in regular medium or in starvation medium. Bar, 10 μm.

Source data are provided as a Source data file.

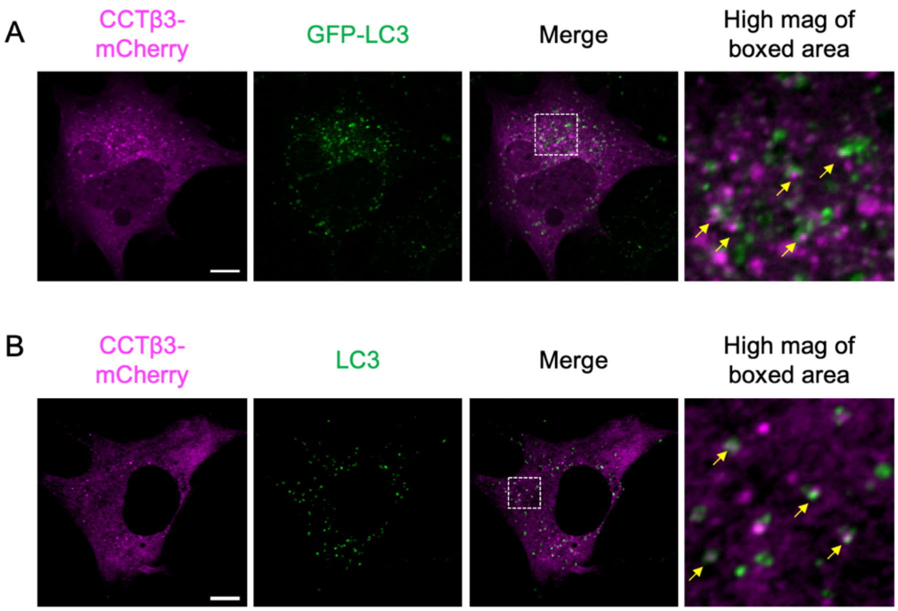

Supplementary Fig. 3. CCTβ3 in MEFs

(A) Distribution of CCTβ3-mCherry (magenta) and GFP-LC3 (green) in MEFs treated for 1 h with 0.25 μM Torin1 in regular medium. Bar, 10 μm.

(B) Distribution of CCTβ3-mCherry (magenta) and endogenous LC3 (green) in MEFs starved for 1 h. Bar, 10 μm.

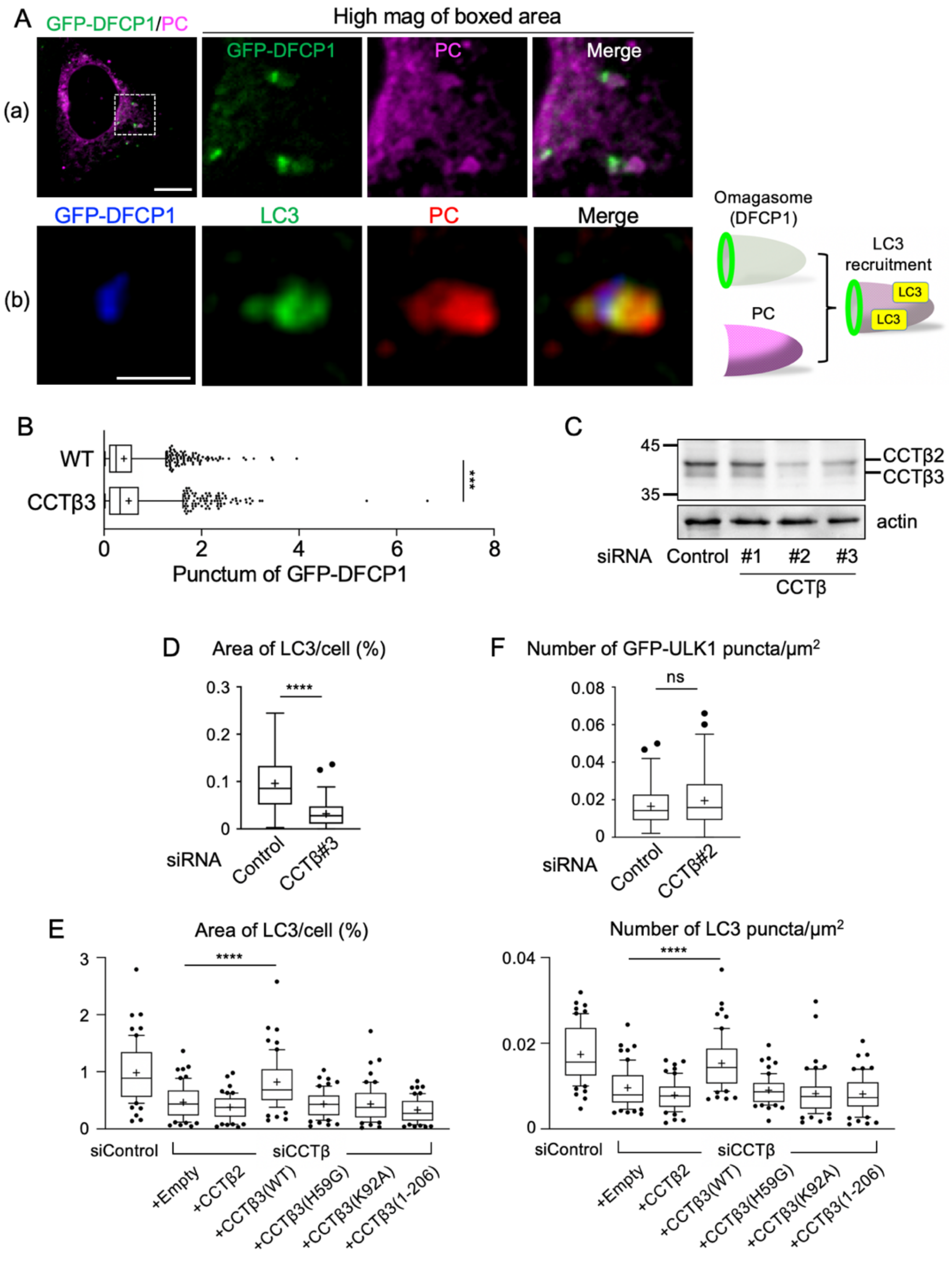

Supplementary Fig. 4. MEFs starved for 8 h

(A) Distribution of *de novo*-synthesized PC, GFP-DFCP1, and endogenous LC3 in MEFs starved for 8 h. Propargylcholine (0.25 mM) was added only for the last 1 h. (a) PC (magenta) and GFP-DFCP1 (green). Bar, 10 μm. (b) PC (red), GFP-DFCP1 (blue), and LC3 (green). The diagram shows the correlation of the three labels and the putative omegasome structure. Bar, 2 μm.

(B) The punctum size of GFP-DFCP1 in MEFs with or without CCTβ3 overexpression. Pooled data from three independent experiments. The number of puncta counted: 1534 (WT) and 1591 (CCTβ3 overexpression). ***$p = 0.0002$ (two-tailed Mann-Whitney test).

(C) Western blotting of CCTβ. MEFs were transfected with either control siRNA or one of three siRNAs targeted to sequences that are common to CCTβ2 and CCTβ3. MEFs treated with siRNA#2 were used except in Supplementary Fig. 4D. Western blotting for actin is shown as a loading control.

(D) The effect of CCTβ siRNA #3 in MEFs starved for 8 h. The total area of immunolabeled LC3 puncta (n = 60). ****$p < 0.0001$ (two-tailed Mann-Whitney test).

(E) Rescue experiment after CCTβ RNAi. MEFs transfected with cDNAs for mCherry alone, mCherry-tagged CCTβ2, CCTβ3, and three CCTβ3 mutants (H59G, K92G, 1-206) were compared (n = 60). ****$p < 0.0001$ (two-tailed Mann-Whitney test).

(F) The effect of CCTβ knockdown on the number of GFP-ULK1 puncta in MEFs starved for 8 h. Pooled data from three independent experiments (n = 90). Two-tailed Mann-Whitney test.

In box plots, the center line indicates the median, box boundaries indicate the 25th and 75th percentiles, whiskers are Tukey-type, and the average is marked as +. Source data are provided as a Source data file.

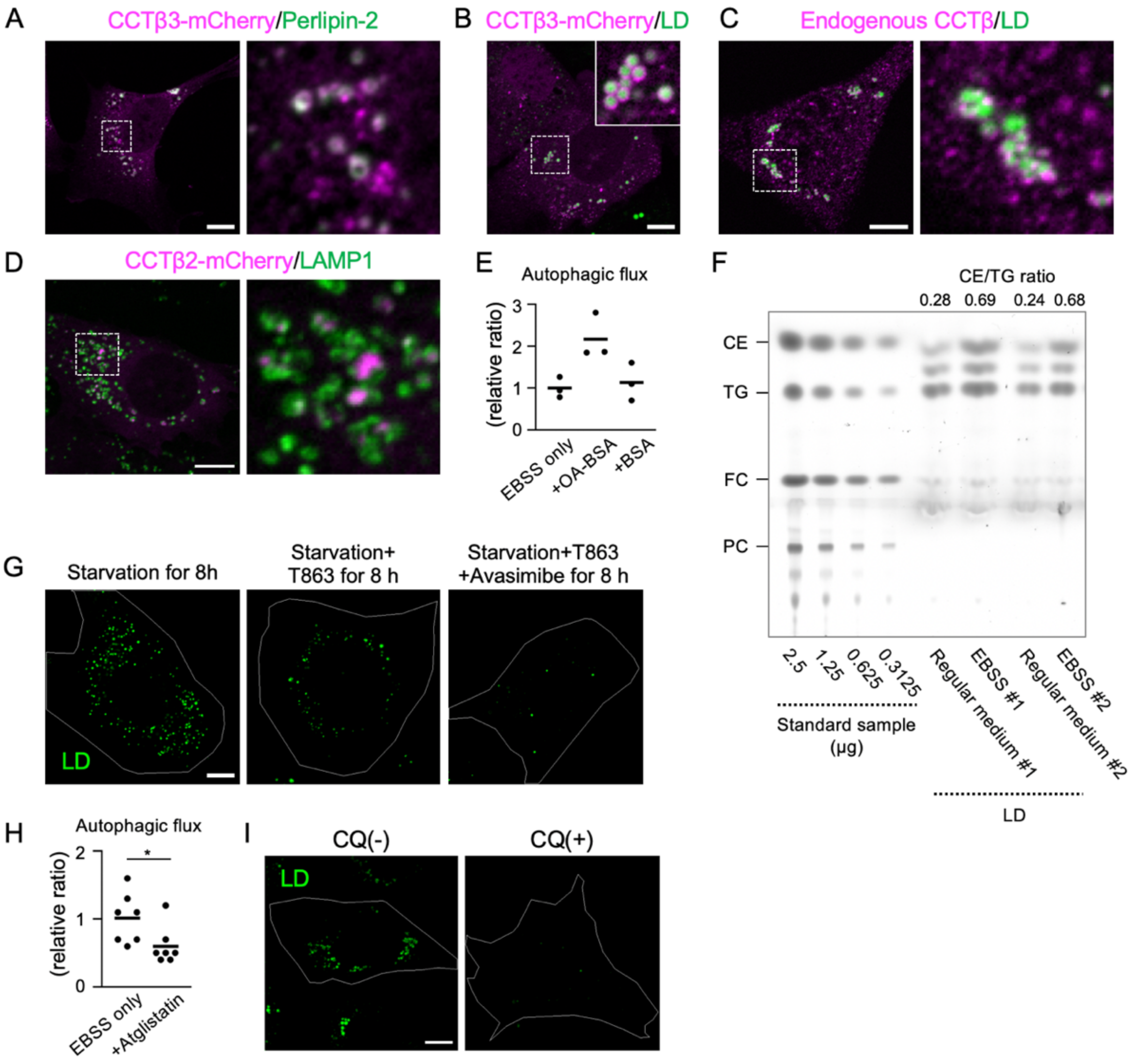

Supplementary Fig. 5. MEFs in prolonged autophagy

(A) CCTβ3-mCherry (magenta) and perilipin-2 (green) in MEFs starved for 8 h. Bar, 10 μm

(B) CCTβ3-mCherry (magenta) and LDs (green) in MEFs treated with 0.25 μM Torin1 for 4 h. Bar, 10 μm.

(C) Endogenous CCTβ (magenta) and LDs (green) in MEFs starved for 8 h. Bar, 10 μm.

(D) CCTβ2-mCherry (magenta) and endogenous LAMP1 (green), a marker for late endosome/lysosomes, in MEFs starved for 8 h. Bar, 10 μm.

(E) The autophagic flux in MEFs cultured for 8 h in EBSS, EBSS containing the OA-BSA complex, and EBSS containing BSA alone.

(F) Thin layer chromatography. LDs were obtained from MEFs cultured in OA-containing regular medium for 1 day or from MEFs cultured for 8 h in OA-containing EBSS. A standard lipid mixture made of cholesterol ester (CE), triglyceride (TG), free cholesterol (FC), and phosphatidylcholine (PC) was run in four different amounts. CE and TG in LD samples were quantified using a calibration curve obtained by the spot density of standard samples.

(G) The effect of 20 μM T863 alone and a mixture of 20 μM T863 and 1 μM avasimibe on LD formation in MEFs starved for 8 h.

(H) The autophagic flux in MEFs cultured for 8 h in EBSS alone or EBSS containing 50 μM Atglistatin. *p = 0.0186 (two-tailed Mann-Whitney test).

(I) The effect of chloroquine (CQ) on LD formation in MEFs. Cells were cultured in EBSS for 8 h with or without 20 μM chloroquine for the last 4 h. Bar, 10 μm.

Source data are provided as a Source data file.

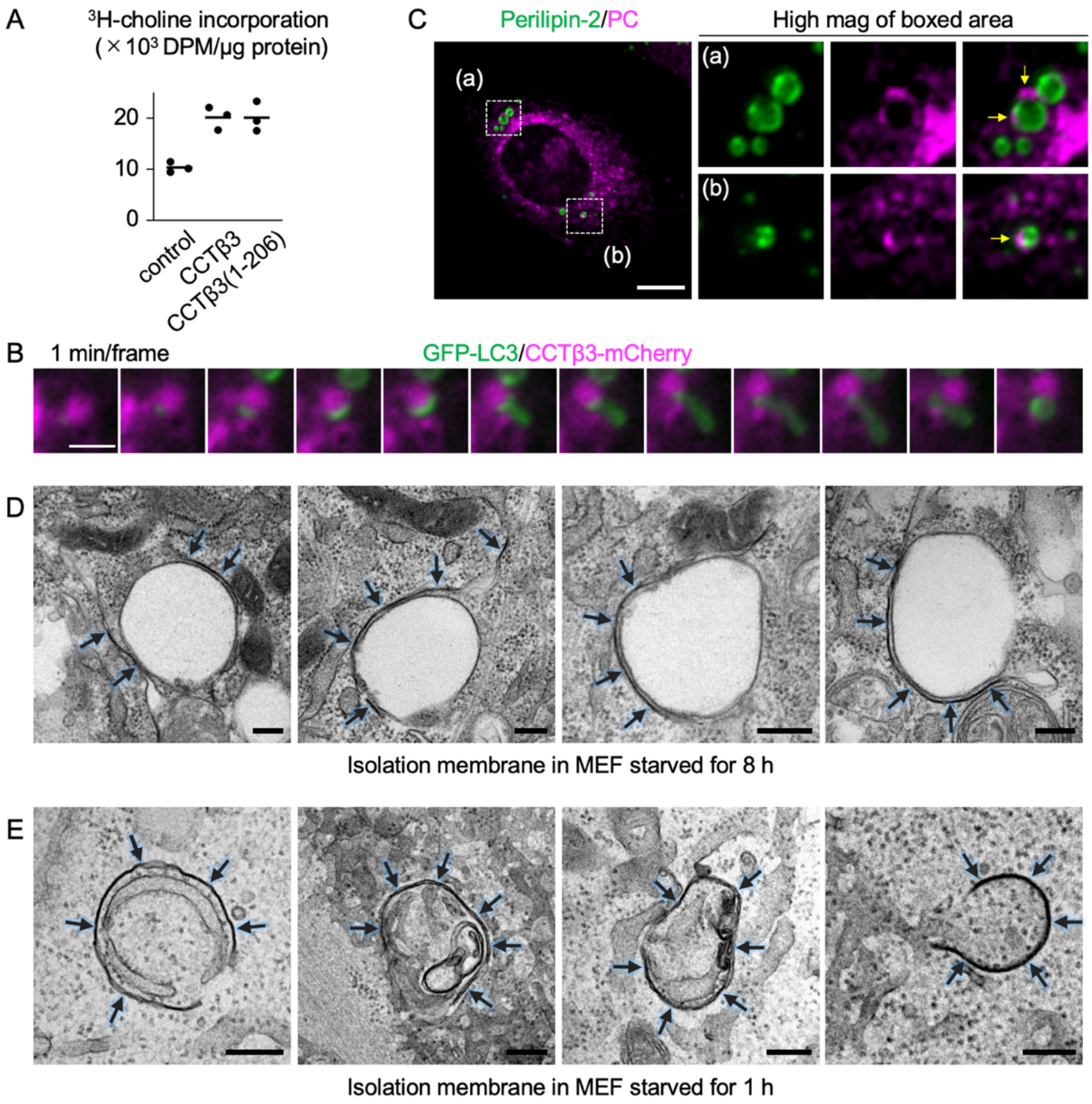

Supplementary Fig. 6. PC and isolation membranes in MEFs

(A) PC synthetic activity. MEFs overexpressing CCTβ3 or CCTβ3(1-206) were compared with untransfected MEFs. Cells were cultured for 8 h in starvation medium and ^{3}H-choline was added for the last 30 min. A representative result of two independent experiments.

(B) Formation and elongation of isolation membranes/autophagosomes (green, GFP-LC3) from LDs where CCTβ3-mCherry (magenta) accumulates. Selected images from Supplementary Movie 3. Bar, 2 μm.

(C) *De novo*-synthesized PC (magenta) and endogenous perilipin-2 (green) in MEFs starved for 8 h. Propargylcholine was added to the medium only for the last 1 h. Intense PC labeling is marked by arrows. Bar, 10 μm.

(D) EM of MEFs overexpressing CCTβ3 and starved for 8 h. Electron-dense isolation membranes (arrows) adhere to LDs. Bars, 0.2 μm.

(E) EM of MEFs overexpressing CCTβ3 and starved for 1 h. Isolation membranes (arrows) are adjacent to the ER. Bars, 0.2 μm.

Source data are provided as a Source data file.

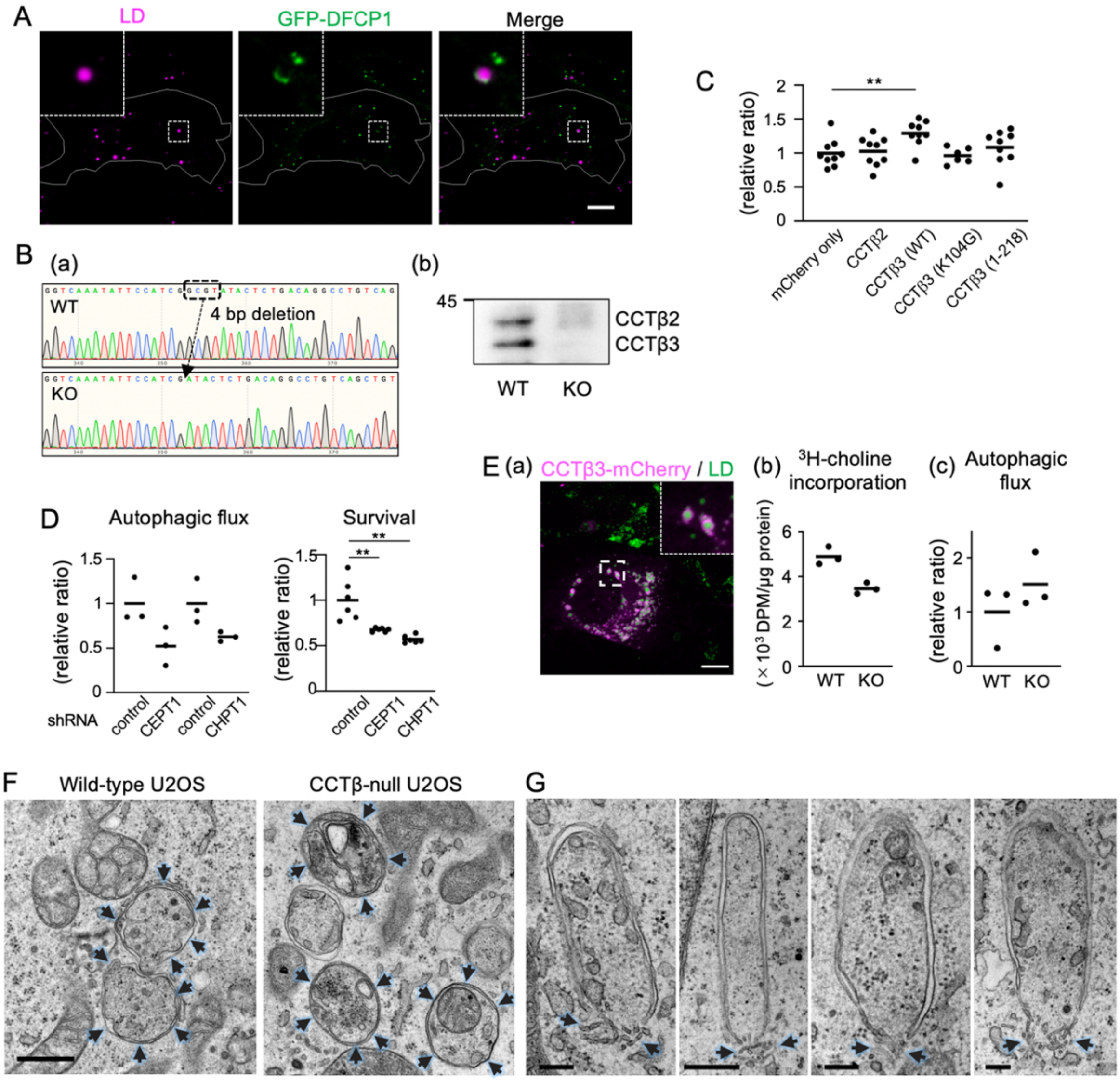

Supplementary Fig. 7. U2OS starved for 24 h

(A) Distribution of GFP-DFCP1 (green) and LDs (magenta) in U2OS starved for 24 h. Bar, 10 μm.

(B) CCTβ-null U2OS. (a) Deletion of 4 bp in the target genome DNA was verified by sequencing. (b) Western blotting by anti-CCTβ antibody.

(C) Cell survival. CCTβ-null U2OS transfected with cDNAs for mCherry alone, mCherry-tagged CCTβ2, CCTβ3, and two CCTβ3 mutants (K104G and 1-218) was cultured as shown in Fig. 7D. The cell numbers after three days of starvation were compared. Pooled data from two independent experiments [n = 6 for CCTβ3(1-218); n = 9 (for the others)]. *p = 0.0132 (two-tailed Mann-Whitney test).

(D) U2OS expressing CEPT1 or CHPT1 shRNA was compared with U2OS expressing control shRNA. A representative result of two independent experiments. For the survival experiment, n = 6, **p = 0.0022 (two-tailed Mann-Whitney test).

(E) CCTβ-null Huh7 starved for 24 h. (i) Distribution of CCTβ3-mCherry (magenta) and LDs (green). Bar, 10 μm. (ii) PC synthetic activity. (iii) The autophagic flux.

(F) Autophagosomes (arrows) in wild-type and CCTβ-null U2OS starved for 24 h. Bar, 0.5 μm.

(G) Elongated isolation membranes in CCTβ-null U2OS starved for 24 h. IMAT membranes are marked by arrows. Bars, 0.2 μm.

Source data are provided as a Source data file.

Supplementary Table 1. Sequences of PCR primers

Species	Protein	Primer sequence (5' – 3')
Human	CCTα	Forward: ggaagatctaccatggatgcacagtgttcagccaagg<br>Reverse: acgcgtcgactggtcttcttcatcctcactgatatc
	CCTβ2	Forward: ggaagatctaccatgccagtagttaccactgatgctg<br>Reverse: acgcgtcgactgcttttcatcctcatccccctcgc
	CCTβ3	Forward: ggaagatctaccatggtaggccatcaggagtgcatcatg<br>Reverse: acgcgtcgactgcttttcatcctcatccccctcgc
Mouse	CCTα	Forward: gaagatctaccatggatgcacagagttcagctaaagtc<br>Reverse: acgcgtcgactggtcctcttcatcctcgctgatgtc
	CCTβ2	Forward: gaagatctaccatgccagtacttaccactgatgc<br>Reverse: acgcgtcgactgcttctcatcctcatcccc
	CCTβ3	Forward: gaagatctaccatggacaaggacgaattct<br>Reverse: acgcgtcgactgcttctcatcctcatcccc
