# Supplementary material for: Long-term autophagy is sustained by activation of CCTβ3 on lipid droplets
Source: Nat Commun. 2020 Sep 8;11:4480. doi: 10.1038/s41467-020-18153-w (PMC7479109; doi:10.1038/s41467-020-18153-w)
Supplement: Supplementary file 2 — Peer Review File [file 41467_2020_18153_MOESM2_ESM.docx]

Peer Review Information

**Journal:** Nature Communications

**Manuscript title:** Long-term autophagy is sustained by activation of CCTβ3 on lipid droplets

**Corresponding author name(s):** Prof Toyoshi Fujimoto

**Editorial notes:**

**none**

**Reviewer comments & decisions:**

**Reviewer comments, first version:**

Reviewers' comments:

Reviewer #1 (Remarks to the Author):

In this manuscript, Ogasawa et al show that nascent autophagic membranes recruit phosphatidylcholine (PC) newly synthesized by the enzyme CTP:phosphocholine cytidylyl-transferase b3 (CCTb3). This is an enzyme that belongs to a family of rate-limiting enzymes for the synthesis of PC in the Kennedy pathway. They found that CCTb3 is recruited to autophagic membranes in the early stage of starvation, but it is then recruited to autophagy-derived lipid droplets (LDs) upon prolonged starvation. Isolation membrane biogenesis appears to occur in the vicinity of LDs. The authors also found that silencing and overexpressing CCTb3 decreases and increases autophagy flux during prolonged starvation, respectively. They also extend their observations to other cell lines, such as U2OS cells. The main conclusion of this manuscript is that PC synthesis mediated through CCTb3 on LDs is necessary to sustain autophagy and cell survival during prolonged starvation.
This is an interesting manuscript that capitalizes on a powerful and elegant technology (i.e., click chemistry) to understand where de novo PC synthesis occurs during nutrient deprivation. The conclusions of this manuscript overlap perhaps a little too much with a recent manuscript published in Autophagy (Andrejeva et al), which should be better integrated into the current manuscript as well as compared/contrasted. The novelty unfortunately takes a hit, although the focus of this manuscript under consideration is on a different CCT isoform and there is a LD angle that is not covered by Andrejeva et al. Given this overlap, this referee feels that additional work is required to make the current manuscript more comprehensive and potentially worthy of publication.
- There is no data indicating that the catalytic activity of CCTb3 is required to sustain autophagy. The authors should attempt to rescue the knockdown or knockout of CCTb3 with catalytically active and inactive constructs
- The authors focus on a specific enzyme (CCTb3) but the pathways leading to PC synthesis are very well defined. Does inhibition of choline kinase replicate the CCTb3 KD findings?
- What are the steps involved in PC synthesis from LDs? Is LD droplet hydrolysis required to generate PC via CCTb3? Can the authors demonstrate this?
- Is CCTb3 dispensable to sustain autophagy in cells that express phosphatidylethanolamine N-methyltransferase (PEMT), such as hepatocytes?


Reviewer #2 (Remarks to the Author):

The authors of this study present evidence that the CCTb3 isoform expressed in murine cells is involved in production of PC needed for membrane biogenesis during autophagy. Using MEFs, they show that CCTb3 is recruited to the isolation membrane during starvation-induced autophagy, and with prolonged starvation appears on LDs. A combination of CCT knockdown and overexpression is used to show that CCTB3 is required to maintain autophagic flux in 8 h starved cells, while there is minimal effect in cells that are starved for 2 h. Since CCTb3 is the rate-limiting enzyme for PC synthesis, it follows that recruitment to isolation membranes and autophagy-derived LDs is driving the localized production of CDP-choline for synthesis of PC by the terminal enzyme that have been suggested to also locate at these sites.

The study is interesting and novel for describing a mechanism for coordinating the synthesis of PC for membranes required for autophagy. As well, CCTb3 is a poorly described isoform of the CCT gene family whose function is unknown. The imaging and quantitation of localized PC synthesis with autophagy is impressive. Some major and minor concerns with the study are listed below.
1. It is clear that autophagic flux is not affected by altered CCTb3 during short term starvation but the conclusion that it has a role in long-term starvation is rather weak. Fig. 4D and G seems to show very minor effects of over-expression and knockdown on flux. The authors should repeat the knockdown experiments with another siRNA to validate this point, as well as other data in this figure that rely on only one siRNA.

2. The discussion mentions that all the starvation experiments are done in choline-free conditions. This raises the question as to whether choline depletion alone is causing CCTb3 to localize to isolation membrane sites and LDs during starvation of MEFs. Is this just a choline-depletion response due to PC-deficiency at sites of autophagy or is CCTB3 recruited as part of the autophagy complex? A simple experiment would be to add choline to the starvation medium to see if CCTB3 recruitment is affected. Choline could possibly drive PC synthesis by other CCT isoforms.

3. The authors should define the differences between the CCTb isoforms as well as the differences between human and mouse isoforms. It seems that the murine CCTb2 and CCTb3 isoforms are now referred to as CCTb1 and CCTb2 in the NCBI genome data base. As well, there are three human CCTb isoforms and it is unclear which is a homologue of the murine CCTb3?

4. Is CCTb3 recruited to isolation membranes or LDs when autophagy is activated by other mechanisms such as mTORC inhibition or knockdown?

5. In the methods section it is stated that CCTb was knocked out in MEFs using CRISPR but all the figures indicate that siRNAs were used. Likewise, it is stated that CCTb was knocked out in U2OS cells but there is no indication how this was done or any data to show that the enzyme was indeed knocked out. Without this information it is unclear how most of the experiments were done.

6. Figure 7 is problematic for several reasons. First, (and as mentioned above) CCTb is said to be knocked out but there is not indication of how this was done or evidence that it was successful. Second, it is unclear if U2OS cells even express CCTb or, if so, which isoforms are expressed (ie. need a blot). Third, the murine CCTb3 isoform are used to ‘rescue’ the KO when they should use the human homologue. Again it is unclear whether a CCTb3 homologue exists in human cells.

7. Figure 4C needs a load control to get a sense of the degree of silencing or KO.


Minor points
1. Difficult to see that cholesterol ester to TG ratio is affected in suppl Fig. 5D.

2. Based on labeling with propargylcholine, it is proposed that de novo synthesized PC is preferentially and rapidly incorporated into autophagosomes, which is sort of contrary to a recent report (as noted by the authors). Their conclusion largely derives from pulse labeling Fig. 1D and Figure 2. The interpretation of the data in Fig. 1D is concerning since the incorporation of propargylcholine is done under different conditions for the ‘de novo’ versus ‘existing’ PC. The ‘existing’ PC is labeled in regular medium that has choline so the incorporation will be reduced by competition, while the de novo is in starvation medium that has no choline thus incorporation of propargylcholine will be greater. As well, some of the labelled PC will have exited the ER during the initial 1h pulse in regular medium. Less propargylcholine incorporation could reduce the absolute signal in puncta in regular medium conditions. The second point relates to Fig. 2 where PC labelling density is compared to membranes with potentially low rates of PC incorporation; the PM will be low since it has to get PC from the ER and the NE is also not a good comparator. It might be technically challenging but the best comparison would be other processes that require membrane biogenesis, such as formation of Golgi export vesicles at ER exit sties. The fact that another report showed that propargylcholine is incorporated into autophagosomes by longer term labeling indicates that the probe is influenced by other variables like choline that are not well controlled.


Reviewer #3 (Remarks to the Author):

The authors show that during starvation cells incorporate de novo synthesized phosphatidylcholine (PC) into autophagic membranes to sustain autophagy and extend cell survival. The novel observation is that CCTβ3, an isoform of the rate-limiting enzyme in the de novo PC synthesis pathway, is enriched in lipid droplets and responsible for PC synthesis after prolonged starvation. The authors make a logical connection to previous works by other that showed autophagy provides lipids to lipid droplets during starvation. Based on this manuscript, there appears to be a cycling of lipids between lipid droplets and the autophagic machinery cycle in cells to cope with starvation. Clinical relevance is proposed in that the dependence of autophagy on CCTβ3 may represent a previously unpredicted vulnerability of some cancers that depend on autophagy for survival. The manuscript reports new and important findings, but some issues remain for clarification, explanation, or modification.

• In the fig1B and fig3B, some PC puncta are not colocalized with Atg proteins. How significant is the percentage of these non-colocalizing puncta? What are the authors’ thoughts about what the non-colocalizing puncta represent?
• Based on fig1D, the authors conclude that “PC existing in organelle membranes may be used for autophagic membrane formation, but in a lower amount than de nov-synthesized PC.” This conclusion would hold only if one assumes that the labelling efficiencies in both experimental conditions are the same, but there are no data presented to support that assumption. The starvation medium with propargylcholine as a sole source of choline likely support more efficient Click labeling for PC than the regular medium, in which propargylcholine may be diluted with other choline sources. The authors should either explain why labeling efficiency must be the same under the 2 conditions or provide supporting data.
• Based on fig2, the authors state “autophagic membrane preferentially incorporate newly synthesized PC”. However, the data only demonstrate that there is more labeling, but didn’t determine the contribution of de novo PC vs. existing PC on organelle membranes. The denser labeling could be a result of more active membrane synthesis rather than preferences for the choline source.
• In Fig4F, did CCTβ3 siRNA change the number of GFP-LC3 puncta as well as total area, or were the number of puncta the same, only smaller? This approaches the question of whether CCTβ3 is involved in de novo PC synthesis for autophagosome initiation or for elongation. If it is elongation CCTβ3 is involved, then CCTβ siRNA might not affect the number of GFP-LC3 and initiation markers such as ULK1.
• In the fig5B(c), how was OA delivered in medium? Was OA conjugated to BSA? If so, did BSA in the starvation medium affect autophagy? The authors state “this result confirmed the importance of LD to sustain autophagy.” Are lipid droplets truly required or can OA rescue the decline of autophagic flux in the fig5E after 8 hours of starvation? Does pharmacological inhibition of lipolysis affect autophagy during starvation?
• In the fig7C, the differences between wild type and knockout cell line in terms of cell survival might not be due to the differential expression of CCTβ3. It would be more convincing to show the viability of the ko U2OS cells in the setting of re-expression of wild type CCTβ, as well as the mutant (1-206) and control. The viability associated with CCTβ3 might not derived from its function in de novo PC synthesis for autophagy, so the inhibitors or siRNA targeting its immediate downstream effectors in this pathway such as CEPT1 and CPT1 can be employed for confirmation.
• In the supplemental fig6C, the green staining fills the lipid droplets, which is more consistent with Bodipy rather than Perilipin-2 staining, which usually is on the periphery of the LDs. Is this figure labeled correctly? In any event, the authors should not rely soley on immunofluorescence staining of Perilipin-2 to identify lipid droplets. In at least one definitive experiment, they should confirm colocalization of CCTβ3 and of LC3 or other autophagy markers with lipid droplets by a biochemical method, such as western blotting of isolated lipid droplets.
• Do the authors have any insight as to why CCTβ3 (1-206) does not localize to lipid droplets and the basis for its being “constitutively active” as they state? The authors think that CCTβ3 must be on lipid droplets to be activated for the purpose of loading autophagic membranes with PC. In this context, what is the model for that activation? A post-translational modification? A conformational change? A protein-protein interaction? Binding a cofactor? In other words, what is known about the “activation” of CCTβ3?

**Author rebuttal, first version:**

Reviewer #1:

*In this manuscript, Ogasawa et al show that nascent autophagic membranes recruit phosphatidylcholine (PC) newly synthesized by the enzyme CTP:phosphocholine cytidylyl-transferase b3 (CCTb3). This is an enzyme that belongs to a family of rate-limiting enzymes for the synthesis of PC in the Kennedy pathway. They found that CCTb3 is recruited to autophagic membranes in the early stage of starvation, but it is then recruited to autophagy-derived lipid droplets (LDs) upon prolonged starvation. Isolation membrane biogenesis appears to occur in the vicinity of LDs. The authors also found that silencing and overexpressing CCTb3 decreases and increases autophagy flux during prolonged starvation, respectively. They also extend their observations to other cell lines, such as U2OS cells. The main conclusion of this manuscript is that PC synthesis mediated through CCTb3 on LDs is necessary to sustain autophagy and cell survival during prolonged starvation.*

*This is an interesting manuscript that capitalizes on a powerful and elegant technology (i.e., click chemistry) to understand where de novo PC synthesis occurs during nutrient deprivation. The conclusions of this manuscript overlap perhaps a little too much with a recent manuscript published in Autophagy (Andrejeva et al), which should be better integrated into the current manuscript as well as compared/contrasted. The novelty unfortunately takes a hit, although the focus of this manuscript under consideration is on a different CCT isoform and there is a LD angle that is not covered by Andrejeva et al. Given this overlap, this referee feels that additional work is required to make the current manuscript more comprehensive and potentially worthy of publication.*

We are very happy to know that you found our work interesting. We also appreciate that you read the manuscript carefully, acknowledged the originality of our findings, and made additional suggestions to improve this paper. We conducted several new experiments and revised the manuscript significantly. We also discussed clearly how our work differs from that of Andrejeva et al. (page 12, 1st paragraph).

*- There is no data indicating that the catalytic activity of CCTb3 is required to sustain autophagy. The authors should attempt to rescue the knockdown or knockout of CCTb3 with catalytically active and inactive constructs.*

We appreciate that you pointed out this critical issue. We did the rescue experiment using wild-type CCTβ3, CCTβ2, and CCTβ3 mutants that are defective either in the catalytic activity or membrane binding. The result shows that autophagy in prolonged starvation is rescued only by wild-type CCTβ3, but not by the others, including CCTβ3 (1-206), which is constitutively active but shows no LD binding. Our results support the conclusion that CCTβ3, which is activated by binding to LDs, is indispensable in sustaining autophagy in long-starved cells (page 7, last paragraph; Supplementary Fig. 4E) .

*- The authors focus on a specific enzyme (CCTb3) but the pathways leading to PC synthesis are very well defined. Does inhibition of choline kinase replicate the CCTb3 KD findings?*

Thank you for raising this interesting point. Involvement of Kennedy pathway enzymes other than CCTs, i.e., choline kinase, [choline/ethanolamine phosphotransferase](https://en.wikipedia.org/wiki/Diacylglycerol_cholinephosphotransferase)1 (CEPT1), and choline phosphotransferase1 (CHPT1), was examined. With regards to choline kinase, its knockdown did not significantly affect the autophagic flux. This result agrees with that of Andrejeva et al. (2019), who examined the effect of choline kinase knockdown on autophagy in a different setting. In contrast, knockdown of either CEPT1 or CHPT1 significantly suppressed autophagy and cell survival in a starvation medium, confirming the importance of PC synthesis in prolonged autophagy. This result was incorporated into the revised manuscript (page 10, 3rd paragraph; Supplementary Fig. 7D).

*- What are the steps involved in PC synthesis from LDs? Is LD droplet hydrolysis required to generate PC via CCTb3? Can the authors demonstrate this?*

Thank you very much again for pointing out an important issue. We blocked LD hydrolysis with Atglistatin, a selective ATGL inhibitor, and found that autophagy in MEF starved for 8 h was downregulated. On the other hand, from an experiment performed based on a comment from another referee, we found that the addition of oleic acid to a starvation medium upregulates autophagy. These data suggest that free fatty acids liberated from LDs by ATGL may be involved in the autophagosome formation in long-starved cells. The result was incorporated into the revised manuscript (Supplementary Fig. 5H; page 8, last paragraph).

*- Is CCTb3 dispensable to sustain autophagy in cells that express phosphatidylethanolamine N-methyltransferase (PEMT), such as hepatocytes?*  

Thank you very much for the insightful question. To examine this point, we used Huh7, a hepatocarcinoma cell line. In Huh7, CCTβ3 is recruited to LDs in prolonged starvation, as in MEF and U2OS, and the depletion of CCTβ suppressed PC synthesis. Nevertheless, autophagy was not decreased in CCTβ-null Huh7 in comparison to wild-type Huh7 (Supplementary Fig. 7E). This result indicates that the contribution of CCTβ3 is not crucial in Huh7, suggesting that PC synthesis by the PEMT pathway may be sufficient for sustaining autophagy in hepatocytes (page 10, last paragraph).

Reviewer #2:

*The authors of this study present evidence that the CCTb3 isoform expressed in murine cells is involved in production of PC needed for membrane biogenesis during autophagy. Using MEFs, they show that CCTb3 is recruited to the isolation membrane during starvation-induced autophagy, and with prolonged starvation appears on LDs. A combination of CCT knockdown and overexpression is used to show that CCTB3 is required to maintain autophagic flux in 8 h starved cells, while there is minimal effect in cells that are starved for 2 h. Since CCTb3 is the rate-limiting enzyme for PC synthesis, it follows that recruitment to isolation membranes and autophagy-derived LDs is driving the localized production of CDP-choline for synthesis of PC by the terminal enzyme that have been suggested to also locate at these sites.  

The study is interesting and novel for describing a mechanism for coordinating the synthesis of PC for membranes required for autophagy. As well, CCTb3 is a poorly described isoform of the CCT gene family whose function is unknown. The imaging and quantitation of localized PC synthesis with autophagy is impressive. Some major and minor concerns with the study are listed below.*

Thank you for your kind words. We are particularly pleased to learn that you found our study interesting and novel and wrote that our imaging method was impressive. We also thank you for reading the manuscript carefully and for providing us with many constructive comments. We conducted several new experiments and revised the manuscript significantly.

*- It is clear that autophagic flux is not affected by altered CCTb3 during short term starvation but the conclusion that it has a role in long-term starvation is rather weak. Fig. 4D and G seems to show very minor effects of over-expression and knockdown on flux. The authors should repeat the knockdown experiments with another siRNA to validate this point, as well as other data in this figure that rely on only one siRNA.*

Thank you for pointing out this critical issue. In order to validate the results obtained by CCTβ knockdown, we performed rescue experiments by re-expression of wild-type CCTβ3, CCTβ2 or CCTβ3 mutants defective either in the catalytic activity or membrane/LD binding, and found that the autophagic suppression by CCTβ depletion is rescued by wild-type CCTβ3 but not by the others (Supplementary Fig. 4E). We also confirmed that transfection of another siRNA for CCTβ (i.e., CCTβ siRNA #3) suppressed autophagy (Supplementary Fig. 4D). These results corroborated that CCTβ3 is critical for maintaining autophagy in prolonged starvation.

*- The discussion mentions that all the starvation experiments are done in choline-free conditions. This raises the question as to whether choline depletion alone is causing CCTb3 to localize to isolation membrane sites and LDs during starvation of MEFs. Is this just a choline-depletion response due to PC-deficiency at sites of autophagy or is CCTB3 recruited as part of the autophagy complex? A simple experiment would be to add choline to the starvation medium to see if CCTB3 recruitment is affected. Choline could possibly drive PC synthesis by other CCT isoforms.*

We appreciate that you proposed an experiment to test whether choline depletion alone causes CCTβ3 to distribute in autophagic membranes and LDs. To test this possibility, we first measured the total choline concentration in the regular culture medium (i.e., Dulbecco’s minimum essential medium supplemented with 10% fetal bovine serum) and added the same concentration of choline (62 μM) to the starvation medium (i.e., Earle’s balanced salt solution). Even when cultured in this choline-containing starvation medium, CCTβ3 was recruited to the isolation membrane/autophagosome and LDs as shown below (please compare with Fig. 3B [1 h] and Fig. 5A [8 h]). This result was incorporated into the revised manuscript (page 6, 3rd paragraph; page 8, 2nd paragraph).

1 h

8 h

10 μm

CCTβ3-mCherry/LC3

CCTβ3-mCherry/LD

*- The authors should define the differences between the CCTb isoforms as well as the differences between human and mouse isoforms. It seems that the murine CCTb2 and CCTb3 isoforms are now referred to as CCTb1 and CCTb2 in the NCBI genome data base. As well, there are three human CCTb isoforms and it is unclear which is a homologue of the murine CCTb3?* 

We apologize that we did not clearly define the nomenclature of CCTβ isoforms in the original manuscript, especially since the nomenclature of CCT isoforms is actually rather confusing. In the NCBI website, CCTβ2 protein is annotated as choline-phosphate cytidylyltransferase B isoform 1 (NP997593.1 [mouse], NP004836.2 [human]), whereas CCTβ3 protein is annotated as isoform 2 (NP808214.1 [mouse], NP001156736.1 [human]). In contrast, in the Universal Protein Resource (UniProt) database, human CCTβ2 and CCTβ3 proteins are listed as isoform 2 and isoform 3, respectively. Because of this discrepancy, we think it appropriate to use the protein names that were assigned by the papers that reported these isoforms for the first time (human CCTβ2 by Lykidis et al, JBC 274, 26992, 1999; mouse CCTβ2 and CCTβ3 by Karim et al, BBA 163, 1, 2003). Both CCTβ2 and CCTβ3 proteins are highly similar between mouse and human (> 98% identity). The CCTβ isoforms in mouse and human cells are clearly defined in the revised manuscript (page 6, 3rd paragraph [mouse]; page 10, 3rd paragraph [human]).

*- Is CCTb3 recruited to isolation membranes or LDs when autophagy is activated by other mechanisms such as mTORC inhibition or knockdown?* 

To address this question, the distribution of CCTβ3 was examined in MEFs treated with 0.25 μM Torin1. In cells treated with Torin1 for 1 h, CCTβ3-mCherry showed recruitment to GFP-LC3 puncta, representing isolation membranes/autophagosomes, and after a longer treatment with Torin1, it was distributed to LDs. These results showed that CCTβ3 undergoes similar redistribution whether autophagy is induced by amino acid starvation or by Torin1 in nutrient-rich conditions. The results were incorporated as Supplementary Figs. 3A and 5B.

*- In the methods section it is stated that CCTb was knocked out in MEFs using CRISPR but all the figures indicate that siRNAs were used. Likewise, it is stated that CCTb was knocked out in U2OS cells but there is no indication how this was done or any data to show that the enzyme was indeed knocked out. Without this information it is unclear how most of the experiments were done.* 

We apologize for the confusion that was caused by the insufficient description in the original manuscript. All the experiments in MEFs were performed using siRNAs, whereas those in U2OS used a CCTβ-null cell line generated by the CRISPR/Cas9 method. In the revised manuscript, we described the method to generate CCTβ-null U2OS (page 16, the “Gene knockout by CRISPR-Cas9 system” section). We also presented the results of genome DNA sequencing and Western blotting, which validated that the gene knockout was accomplished successfully (Supplementary Fig. 7B).

*- Figure 7 is problematic for several reasons. _1)_ First, (and as mentioned above) CCTb is said to be knocked out but there is not indication of how this was done or evidence that it was successful. _2)_ Second, it is unclear if U2OS cells even express CCTb or, if so, which isoforms are expressed (ie. need a blot). _3)_ Third, the murine CCTb3 isoform are used to ‘rescue’ the KO when they should use the human homologue. Again it is unclear whether a CCTb3 homologue exists in human cells.*

We apologize again for the confusion due to the insufficiency of the original manuscript.

1) As we mentioned in our response to the previous comment, the method to generate the CCTβ-null U2OS (page 16, the “Gene knockout by CRISPR-Cas9 system” section) and the results validating the gene knockout were incorporated in the revised manuscript (Supplementary Fig. 7B).
2) The results of Western blotting using anti-CCTβ antibody were added (Supplementary Fig. 7B). U2OS expresses both CCTβ2 and CCTβ3.
3) The rescue experiment in U2OS (Fig. 7B) was performed by transfecting cDNAs encoding human CCTβ proteins. The use of human cDNAs was clearly described in the revised manuscript (page 10, 3rd paragraph).

*- Figure 4C needs a load control to get a sense of the degree of silencing or KO.* 

The result of Western blotting for actin was added to Supplementary Fig. 4C as a loading control.

*Minor points*

*- Difficult to see that cholesterol ester to TG ratio is affected in suppl Fig. 5D.*

We apologize that the original manuscript did not present the numerical data obtained from the TLC result. Calibration curves were prepared based on the spot densities of standard samples and used to estimate the amount of triglyceride and cholesterol ester in the LD samples, which were loaded in two different amounts. The obtained cholesterol ester-to-triglyceride ratio was significantly higher in LDs obtained from long-starved MEFs than in LDs from MEFs cultured in oleic acid-containing regular medium. These results are presented in Supplementary Fig. 5F.

*- Based on labeling with propargylcholine, it is proposed that de novo synthesized PC is preferentially and rapidly incorporated into autophagosomes, which is sort of contrary to a recent report (as noted by the authors). Their conclusion largely derives from pulse labeling Fig. 1D and Figure 2. _1)_The interpretation of the data in Fig. 1D is concerning since the incorporation of propargylcholine is done under different conditions for the ‘de novo’ versus ‘existing’ PC. The ‘existing’ PC is labeled in regular medium that has choline so the incorporation will be reduced by competition, while the de novo is in starvation medium that has no choline thus incorporation of propargylcholine will be greater. _2)_ As well, some of the labelled PC will have exited the ER during the initial 1h pulse in regular medium. Less propargylcholine incorporation could reduce the absolute signal in puncta in regular medium conditions. _3)_The second point relates to Fig. 2 where PC labelling density is compared to membranes with potentially low rates of PC incorporation; the PM will be low since it has to get PC from the ER and the NE is also not a good comparator. It might be technically challenging but the best comparison would be other processes that require membrane biogenesis, such as formation of Golgi export vesicles at ER exit sties. _4)_The fact that another report showed that propargylcholine is incorporated into autophagosomes by longer term labeling indicates that the probe is influenced by other variables like choline that are not well controlled.*

1) We re-examined the PC puncta formation by equalizing the choline concentration in the regular and starvation media at 62 μM. The results were essentially the same as those without choline supplementation to starvation medium (Supplementary Fig. 1F). These results indicate that the difference in the PC puncta formation in the two conditions was not affected by choline.
2) Thank you very much for raising this concern. What can be deduced from the result of Fig. 1D is that *de novo* synthesized PC is used more for autophagic membrane formation than is PC existing in organelle membranes. As pointed out correctly, the latter cannot be restricted to PC in the ER membrane and should include PC in other organelles, such as mitochondria. We revised the text in the revised manuscript to make this point clearer (page 6, 1st paragraph).
3) We apologize that this result was presented in a confusing manner. The data on the plasma membrane were not shown as a comparison group, but were intended to be a reference to confirm the specificity of the method. To avoid confusion, the result on the plasma membrane was removed from the revised manuscript.

With regards to the outer nuclear membrane issue, we respectfully argue that it is continuous with the ER and can be thought of as a proxy for the ER membrane. To validate this, we added a new micrograph as Supplementary Fig. 2D, which shows that PC labeling in the ER membrane is comparable to that in the outer nuclear membrane.

Regarding the ER exit site, we added a set of data, which show that the PC labeling there is equivalent to that of the surrounding ER network, either in regular medium or in starvation medium (Supplementary Fig. 2F).

4) As described above, the preferential incorporation of propargylcholine to autophagic membranes occurs even when choline is present (Supplementary Fig. 1F). The long-term metabolic labeling (6 h) used by Andrejeva et al. (2019) is not appropriate for defining where *de novo* synthesized PC is incorporated because labeled PC becomes a major portion of PC throughout cellular membranes, including cargos engulfed by autophagosomes, which is evident in Fig. 3 of that paper. This methodological issue was discussed in the revised manuscript (page 12, 1st paragraph).

Reviewer #3:

*The authors show that during starvation cells incorporate de novo synthesized phosphatidylcholine (PC) into autophagic membranes to sustain autophagy and extend cell survival. The novel observation is that CCTβ3, an isoform of the rate-limiting enzyme in the de novo PC synthesis pathway, is enriched in lipid droplets and responsible for PC synthesis after prolonged starvation. The authors make a logical connection to previous works by other that showed autophagy provides lipids to lipid droplets during starvation. Based on this manuscript, there appears to be a cycling of lipids between lipid droplets and the autophagic machinery cycle in cells to cope with starvation. Clinical relevance is proposed in that the dependence of autophagy on CCTβ3 may represent a previously unpredicted vulnerability of some cancers that depend on autophagy for survival. The manuscript reports new and important findings, but some issues remain for clarification, explanation, or modification.*

Thank you for your kind words. We are pleased to learn that you considered our findings new and important. We also thank you for reading the manuscript carefully and providing us with many constructive comments. We conducted several new experiments and revised the manuscript significantly.

*- In the fig1B and fig3B, some PC puncta are not colocalized with Atg proteins. How significant is the percentage of these non-colocalizing puncta? What are the authors’ thoughts about what the non-colocalizing puncta represent?*

We thank you for asking this important question. ULK1, WIPI1, and LC3 were shown to be recruited to the autophagome formation site at different stages (Koyama-Honda et al., Autophagy 9, 1491, 2013), whereas DFCP1 distributes in the omegasome, an ER membrane domain generating isolation membranes (Axe et al., J Cell Biol 182, 685, 2008). These results indicate that each Atg protein represents different stages/kinds of autophagic membranes and that different Atg proteins do not necessarily colocalize each other. This explains why the proportion of PC puncta colocalizing with each Atg protein is relatively small. Actually, when PC, LC3, and DFCP1 were labeled simultaneously, PC puncta colocalizing with LC3 and those colocalizing with DFCP1 were largely separate (Supplementary Fig. 1C). A similar separation was observed between PC/LC3 and PC/WIPI1 puncta. Based on these results, we conclude that a majority of PC puncta represent autophagic membranes. These results were incorporated into the revised manuscript (page 5, 1st paragraph).

*- Based on fig1D, the authors conclude that “PC existing in organelle membranes may be used for autophagic membrane formation, but in a lower amount than de nov-synthesized PC.” This conclusion would hold only if one assumes that the labelling efficiencies in both experimental conditions are the same, but there are no data presented to support that assumption. The starvation medium with propargylcholine as a sole source of choline likely support more efficient Click labeling for PC than the regular medium, in which propargylcholine may be diluted with other choline sources. The authors should either explain why labeling efficiency must be the same under the 2 conditions or provide supporting data.*

To address this question, we first quantified the total choline concentration in a regular medium (Dulbecco’s modified Eagle’s medium + 10% fetal bovine serum) and found it to be 62 μM. By using a starvation medium to which 62 μM choline was added, PC puncta formation was re-examined. The result was essentially the same as that obtained without the choline supplementation: that is, PC puncta were formed more by labeling in starvation medium than by labeling in regular medium followed by 1 h chase in starvation medium (Supplementary Fig. 1F). These results indicate that the PC puncta formation by propargylcholine labeling is not affected by the low level of choline in the culture medium.

*- Based on fig2, the authors state “autophagic membrane preferentially incorporate newly synthesized PC”. However, the data only demonstrate that there is more labeling, but didn’t determine the contribution of de novo PC vs. existing PC on organelle membranes. The denser labeling could be a result of more active membrane synthesis rather than preferences for the choline source.*

We apologize that our wording was not appropriate and caused confusion. The final step of PC synthesis by the Kennedy pathway is catalyzed by two microsomal enzymes, CEPT1 and CHPT1, both of which are transmembrane proteins and are not likely to exist in autophagic membranes. Consistently, PC labeling in a regular medium was most intense in the ER. Notwithstanding, autophagosomes were labeled for newly synthesized PC at a much higher density than in the outer nuclear membrane, a proxy of the ER membrane. This result refutes the possibility that newly synthesized PC is first disseminated throughout the general ER membrane and that a portion of such an ER membrane then turns into the autophagosomal membrane. It rather suggests a mechanism that newly synthesized PC is directly incorporated into autophagosomes without passing through the general ER membrane. This, however, does not exclude the possibility that PC in the ER membrane is also used for the autophagosomal membrane formation. Based on these arguments, we changed the sentence as follows: (the results indicated that) authophagic membranes incorporate newly synthesized PC at a higher density than the ER membrane (page 6, 2nd paragraph; page 12, 1st paragraph).

*- In Fig4F, did CCTβ3 siRNA change the number of GFP-LC3 puncta as well as total area, or were the number of puncta the same, only smaller? This approaches the question of whether CCTβ3 is involved in de novo PC synthesis for autophagosome initiation or for elongation. If it is elongation CCTβ3 is involved, then CCTβ siRNA might not affect the number of GFP-LC3 and initiation markers such as ULK1.*

We thank you for raising this interesting point. By knockdown of CCTβ, the GFP-LC3 puncta were decreased in number as well as in total area (added to Fig. 4F). In contrast, the number of GFP-ULK1 was not affected (Supplementary Fig. 4F), which is consistent with the finding that ULK1 and PC colocalize in a much lower frequency than LC3 and PC (Fig. 1B). These results suggest that *de novo* PC synthesis mediated by CCTβ3 may not be very important for the early phase of autophagy, when ULK1 is recruited to the ER (or its proximity), but is involved in a later stage in which the isolation membranes/autophagosomes form and elongate. The implication of the result was incorporated to the revised manuscript (page 7, last paragraph).

*- _1)_ In the fig5B(c), how was OA delivered in medium? Was OA conjugated to BSA? _2)_ If so, did BSA in the starvation medium affect autophagy? _3)_ The authors state “this result confirmed the importance of LD to sustain autophagy.” Are lipid droplets truly required or can OA rescue the decline of autophagic flux in the fig5E after 8 hours of starvation? Does pharmacological inhibition of lipolysis affect autophagy during starvation?*

1) OA was conjugated with fatty acid-free BSA at the 6:1 molar ratio according to Brasaemle et al. (J Biol Chem 272, 9378, 1997) and was added to the medium at the final OA concentration of 0.4 mM. The method and the citation were added to the manuscript (page 15, 1st paragraph).
2) Addition of the same concentration of BSA alone to the starvation medium did not affect the autophagic flux (Supplementary Fig. 5E).
3) Thank you very much for asking these important questions. We found that the addition of OA increased the autophagic flux in prolonged starvation (Supplementary Fig. 5E). Moreover, inhibition of LD hydrolysis with Atglistatin (a selective ATGL inhibitor) reduced the autophagic flux (Supplementary Fig. 5H). These data suggest that free fatty acids liberated from LDs by ATGL may be involved in the autophagosome formation in long-starved cells. The result was incorporated into the revised manuscript (page 8, last paragraph).

*- In the fig7C, the differences between wild type and knockout cell line in terms of cell survival might not be due to the differential expression of CCTβ3. _1)_ It would be more convincing to show the viability of the ko U2OS cells in the setting of re-expression of wild type CCTβ, as well as the mutant (1-206) and control. _2)_ The viability associated with CCTβ3 might not derived from its function in de novo PC synthesis for autophagy, so the inhibitors or siRNA targeting its immediate downstream effectors in this pathway such as CEPT1 and CPT1 can be employed for confirmation.*

1) We thank you for pointing out this critical issue. In order to validate the results obtained in CCTβ-null U2OS cells, we transfected them with cDNAs of wild-type CCTβ3 as well as wild-type CCTβ2 and CCTβ3 mutants that were defective either in the catalytic activity or membrane binding. The results showed that the survival of CCTβ-null after prolonged starvation was increased significantly by transfection of wild-type CCTβ3 cDNA, but not by transfection of the other cDNAs. The survival ratio of CCTβ-null transfected with wild-type CCTβ3 cDNA did not reach the level of wild-type U2OS, but this is probably because the efficiency of cDNA tranfection is about 30% and the expression of CCTβ3 is not constant during the entire starvation period (3 days). These results were incorporated as Supplementary Fig. 7C.
2) We generated stable U2OS clones harboring shRNA for CEPT1 and CHPT1 (CPT1) and examined the effect on the autophagic flux and the cell survival. Knockdown of either CEPT1 or CHPT1 significantly decreased both the autophagic flux and the cell survival in prolonged starvation (Supplementary Fig. 7D). These results support the conclusion that *de novo* PC synthesis is important for autophagy and cell survival in prolonged starvation.

*- _1)_ In the supplemental fig6C, the green staining fills the lipid droplets, which is more consistent with Bodipy rather than Perilipin-2 staining, which usually is on the periphery of the LDs. Is this figure labeled correctly? _2)_ In any event, the authors should not rely soley on immunofluorescence staining of Perilipin-2 to identify lipid droplets. In at least one definitive experiment, they should confirm colocalization of CCTβ3 and of LC3 or other autophagy markers with lipid droplets by a biochemical method, such as western blotting of isolated lipid droplets.*

1) We apologize for the confusion that was caused by the poor quality of the immunofluorescence micrograph presented in the PDF format. We replaced it with a better figure in the revised manuscript (Supplementary Fig. 6C).
2) We thank you for the proposal to biochemically test the distribution of CCTβ3 and Atg proteins in isolated LDs. We compared the LD fractions obtained from MEFs in a starvation medium and MEFs in a regular medium containing OA. The results showed that CCTβ3 was highly enriched in LDs isolated from starved MEFs, but not in LDs isolated from MEFs in a regular medium. In contrast, LC3 and DFCP1 did not show enrichment in LDs of starved MEFs, indicating that those Atg proteins are not tightly associated with LDs. These results were incorporated as Fig. 5C and discussed in the text (page 8, 3rd paragraph; page 10, 1st paragraph).

*- Do the authors have any insight as to why CCTβ3 (1-206) does not localize to lipid droplets and the basis for its being “constitutively active” as they state? The authors think that CCTβ3 must be on lipid droplets to be activated for the purpose of loading autophagic membranes with PC. In this context, what is the model for that activation? A post-translational modification? A conformational change? A protein-protein interaction? Binding a cofactor? In other words, what is known about the “activation” of CCTβ3?*

According to the literature (e.g., Cornell and Ridgway, Prog Lipid Res 59, 147, 2015; ref ^15^), the catalytic domain in the N-terminal portion of CCT is inhibited by interacting with the C-terminal portion of CCT when the protein is in the soluble phase, and membrane binding causes the enzymatic activation through a conformational change. CCTβ3(1-206) is thought to be constitutive-active because it lacks the C-terminal regulatory (inhibitory) domain

**Reviewer comments, second version:**

Reviewer #1 (Remarks to the Author):

The authors have satisfyingly addressed every single point I have raised with new experiments and new data. The manuscript has now been significantly strengthened.


Reviewer #2 (Remarks to the Author):

All my comments have been addressed, very nice study.

Just a minor comment regarding the role of PEMT in isolation membrane formation (this was raised by reviewer 1 and is now mentioned in the text on page 10). It is known that many heptocarcinoma cell lines are fetal-like/dedifferentiated and have PEMT low activity. Thus the argument on the bottom of page 10 that PEMT somehow sustains autophagy may not be correct if Huh7 cells have lower PEMT activity relative to normal hepatocytes.


Reviewer #3 (Remarks to the Author):

This reviewer appreciates the comprehensive responses of the authors to all reviewer comments. The revised manuscript is particularly enhanced by the rescue experiments, which strengthen the conclusion that CCTb3 activity on lipid droplets is necessary for membrane synthesis during prolonged autophagy.

Perry Bickel

**Author rebuttal, second version:**

Reviewer #1 (Remarks to the Author):

*The authors have satisfyingly addressed every single point I have raised with new experiments and new data. The manuscript has now been significantly strengthened.*

We thank this reviewer very much again for careful reading of the manuscript and all the constructive comments.

Reviewer #2 (Remarks to the Author):

*All my comments have been addressed, very nice study.*

We thank this reviewer very much again for careful reading of the manuscript and all the constructive comments.

*Just a minor comment regarding the role of PEMT in isolation membrane formation (this was raised by reviewer 1 and is now mentioned in the text on page 10). It is known that many heptocarcinoma cell lines are fetal-like/dedifferentiated and have PEMT low activity. Thus the argument on the bottom of page 10 that PEMT somehow sustains autophagy may not be correct if Huh7 cells have lower PEMT activity relative to normal hepatocytes.*

The expression of PEMT in Huh7 cells were confirmed by RT-PCR and Western blotting

(Wong and Chen, J Virol 90, 9075, 2016), and PEMT knockdown in Huh7 was shown to
decrease *P. berghei* infection (Itoe et al, Cell Host Microbe 16, 778, 2014). These results

indicate that PEMT is expressed at a significant level in Huh7 cells.

Reviewer #3 (Remarks to the Author):

*This reviewer appreciates the comprehensive responses of the authors to all reviewer comments. The revised manuscript is particularly enhanced by the rescue experiments, which strengthen the conclusion that CCTb3 activity on lipid droplets is necessary for membrane synthesis during prolonged autophagy.*

*Perry Bickel*

We are very much thankful to Dr. Bickel for all the constructive comments and insightful questions.

**Reviewer comments, third version:**

**Author rebuttal, third version:**
